# Expression of the *Malus sieversii* NF-YB21 Encoded Gene Confers Tolerance to Osmotic Stresses in *Arabidopsis thaliana*

**DOI:** 10.3390/ijms22189777

**Published:** 2021-09-10

**Authors:** Chen Feng, Yanyan Wang, Yueting Sun, Xiang Peng, Xiang Zhang, Xin Zhou, Jiale Jiao, Zefeng Zhai, Yuqin Xiao, Weili Wang, Yang Liu, Tianhong Li

**Affiliations:** State Key Laboratories of Agrobiotechnology, Department of Pomology, College of Horticulture, China Agricultural University, Beijing 100193, China; fengc@cau.edu.cn (C.F.); yabofei1212@163.com (Y.W.); yuetingsun@126.com (Y.S.); rod@cau.edu.cn (X.P.); 18306391375@163.com (X.Z.); zx51522zzwlwlbb@126.com (X.Z.); qingxuedanchen@163.com (J.J.); zhaizefeng@126.com (Z.Z.); S20193172433@cau.edu.cn (Y.X.); WWL0824@foxmail.com (W.W.)

**Keywords:** *Malus sieversii*, nuclear factor Y, *MsNF*-*YB21*, osmotic stress, antioxidant, gene expression

## Abstract

Drought is the main environmental factor that limits the yield and quality of apples (*Malus × domestica*) grown in arid and semi-arid regions. Nuclear factor Ys (NF-Ys) are important transcription factors involved in the regulation of plant growth, development, and various stress responses. However, the function of *NF-Y* genes is poorly understood in apples. Here, we identified 43 *NF-Y* genes in the genome of apples and conducted an initial functional characterization of the apple *NF-Y*. Expression analysis of *NF-Y* members in *M. sieversii* revealed that a large number of *NF-Ys* were highly expressed in the roots compared with the leaves, and a large proportion of *NF-Y* genes responded to drought treatment. Furthermore, heterologous expression of *MsNF-YB21,* which was significantly upregulated by drought, led to a longer root length and, thus, conferred improved osmotic and salt tolerance in *Arabidopsis*. Moreover, the physiological analysis of *MsNF-YB21* overexpression revealed enhanced antioxidant systems, including antioxidant enzymes and compatible solutes. In addition, genes encoding catalase (*AtCAT2*, *AtCAT3*), superoxide dismutase (*AtFSD1*, *AtFSD3*, *AtCSD1*), and peroxidase (*AtPER12*, *AtPER42*, *AtPER47*, *AtPER51*) showed upregulated expression in the *MsNF-YB21* overexpression lines. These results for the *MsNF-Y* gene family provide useful information for future studies on NF-Ys in apples, and the functional analysis of MsNF-YB21 supports it as a potential target in the improvement of apple drought tolerance via biotechnological strategies.

## 1. Introduction

Drought stress can have a detrimental effect on plant reproduction and, thus, crop productivity [1,2]. Upon exposure to drought stress, osmotic stress emerges, which leads to an accumulation of malondialdehyde (MDA) and cellular component (such as membrane lipids) damage, ultimately restricting plant growth [3,4,5]. Plants have developed a series of physiological and molecular strategies to resist and adapt to drought stress [6,7]. Some physiological events have been identified that occur under drought stress, including increasing proline content and improved antioxidant enzyme activity [8,9]. Furthermore, transcriptomic analyses of plants under drought stress have revealed various gene regulatory mechanisms of the physiological responses; transcription factors (TFs) are vital in mediating genetic networks [10,11,12]. Therefore, identification of these TFs can not only clarify the molecular mechanisms of drought responses, but also provide valuable information for breeding drought tolerant crops.

Nuclear factor Y (NF-Y) is an important TF among eukaryotes, acting as a trimer complex comprising three subunits: NF-YA, NF-YB, and NF-YC [13,14]. Previous studies have revealed that the NF-Y complex regulates its target genes by directly binding to a CCAAT element in promoters [15,16]. In humans (*Homo sapiens*) and yeast, each subunit only has one family protein. However, in plants, a large gene family for each subunit has evolved. For example, *Arabidopsis thaliana* has 10 NF-YAs, 13 NF-YBs, and 13 NF-YCs [17], while, in *Glycine max*, there are 59 NF-Ys (including 12 NF-YAs, 32 NF-YBs, and 15 NF-YCs) [18]. With the number of each subfamily in plants continually increasing, it has become difficult to clarify the NF-Ys target genes network, because of its genetic redundancy and functional divergence [19,20]. In the past few decades, researchers have found that this gene family is involved in regulating plant growth and development, including embryogenesis [21,22,23], flowering, and photosynthesis [24,25,26]. More evidence has shown that NF-Ys can play an essential role in responding to abiotic stresses (including drought, salt, and other stresses) [27,28,29,30]. For instance, expression of *AtNF-YA5* was strongly upregulated by drought stress in an abscisic acid (ABA)-dependent manner, and overexpression or knockdown of this gene resulted in a drought-resistant or drought-sensitive phenomenon, respectively [31]. In addition, overexpression of *AtNF-YA2/3/7/10*, *AtNF-YB1,* or *AtNF-YC9* was also proven to enhance the drought tolerance of transgenic plants [32,33,34]. Interestingly, transcriptional analysis of *AtNF-YA2*/*5-*, *AtNF-YB1-*, or *AtNF-YC9-*overexpressing plants showed that each of the *AtNF-Y* genes managed different subsets of genes, indicating that NF-Y controls multiple regulatory pathways to tailor the response to drought stress [32,33,34]. Indeed, overexpression of *ZmNF-YB16* conferred drought tolerance by improving antioxidant enzyme activity and membrane stability [35], while, in *ZmNF-YA3-*overexpressing plants, improved tolerance was linked to the jasmonic acid (JA) signaling pathway [36]. Furthermore, overexpression of *ZmNF-YC14* in *Arabidopsis* enhanced drought resistance by regulating the expression of endoplasmic reticulum (ER) stress response genes [37].

Although many NF-Y family members have been identified in plants (including apple trees [38]), most of these studies have been limited to structural characterization and expression analysis under abiotic stress. The biological functions of the NF-Ys or the molecular properties of these TFs still remain unclear. More importantly, most of the previous research on NF-Ys has focused on model plants, such as *Arabidopsis thaliana* [32,33,34] and *Zea mays* [35,36]; their function in Rosaceae plants, especially in apple trees, still needs to be studied. In this study, we identified 43 NF-Y members in the apple (*Malus × domestica, M. domestica*) genome, analyzed their phylogenetic relationships, and determined their expression patterns in different tissues and in response to drought stress. Moreover, the functional characterization of *MsNF-YB21* in drought response was investigated in *Arabidopsis*.

## 2. Results

### 2.1. Identification and Phylogenetic Analysis of the MdNF-Y Gene Families

In order to identify the complete NF-Y family members in apple genome, we utilized the BLAST and HMMER scanning methods to examine putative apple *NF-Y* genes. After removing the redundant genes and verifying the conserved domains, a total of 43 putative *MdNF-Y* genes were identified, categorized into three groups (nine *MdNF-YA* genes, 22 *MdNF-YB* genes, and 12 *MdNF-YC* genes) (Table S1). To facilitate the analysis, we renamed these genes based on chromosomal position; all the *MdNF-Ys* genes were named consecutively from *MdNF-YA1* to *9*, *MdNF-YB1* to *22*, and *MdNF-YC1* to *12*. The sizes of the MdNF-Ys ranged from 108 (MdNF-YB9) to 358 (MdNF-YA2) AA residues, with molecular weights from 11.91 (MdNF-YB9) to 38.86 kDa (MdNF-YA2), and the theoretical isoelectric point (pI) ranged from 4.78 (MdNF-YB4) to 9.28 (MdNF-YA3). Detailed information regarding the MdNF-Ys is listed in Table S1.

**Figure 1:**
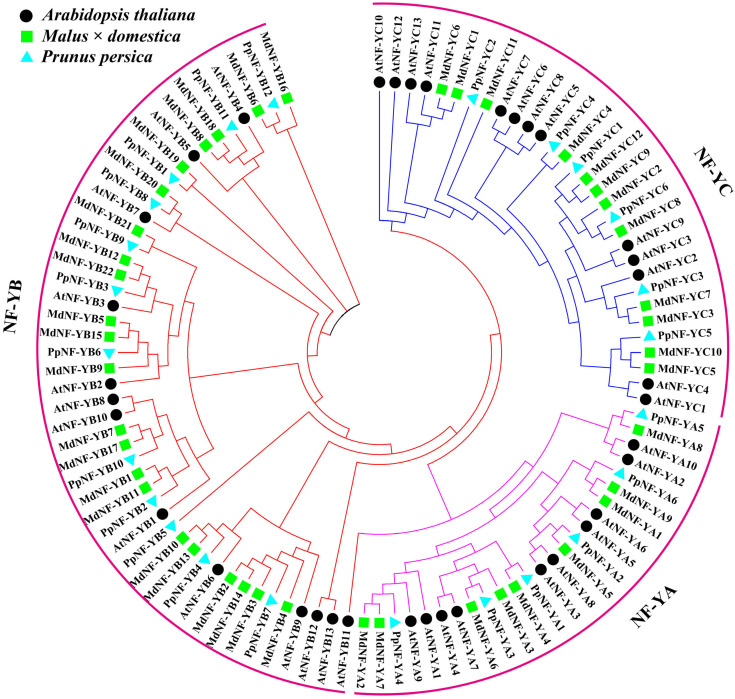
All MdNF-Y, AtNF-Y, and PpNF-Y proteins were clustered into three clades (NF-YA, NF-YB, and NF-YC). The phylogenetic tree of NF-Y TFs was constructed by Molecular Evolutionary Genetics Analysis Version 7.0 software (MEGA7) with the neighbor-joining (NJ) method, using a bootstrap of 1000 replicates.

To further elucidate the evolutionary relationships and the potential functions of the MdNF-Ys, we used MdNF-Y proteins to construct a phylogenetic tree using the MEGA7 Neighbor-Joining method. *Arabidopsis* and *Prunus persic* NF-Y proteins were chosen as reference proteins. As shown in Figure 1, all the NF-Y proteins were divided into three groups, marked by three different colors, corresponding to the three subunit families. Within each subgroup, the NF-Y proteins of apples, peaches, and *Arabidopsis* were phylogenetically closer. Furthermore, each NF-Y subunit family could be clustered into two to six clades in light of their phylogenetic relationships (Figure S1). In terms of the evolutionary relationships, we found that MdNF-YA4/5/8 were the closest to PpNF-YA1/2/5, indicating that these protein pairs may have similar functions. MdNF-YA1/5/9 were clustered with AtNF-YA5, a protein which improves the drought resistance of plants, indicating the possible role of these three MdNF-YA proteins in the drought response.

### 2.2. Chromosomal Location and Duplication Event Analysis

A total of 43 *MdNF-Ys* were unevenly distributed across all 16 apple chromosomes (Figure S2A). Chromosomes 2, 3, 5, 11, and 15 contained the largest number of *MdNF-Y* genes (four), followed by chromosomes 4, 7, 10, and 12, with three, and there were two *MdNF-Y* members each on chromosomes 6, 13, and 14. In addition, chromosomes 1, 16, and 17 contained only one *MdNF-Y* gene.

To analyze the duplication events of the *MdNF-Ys*, we used BioEdit software to calculate the correlation of each *MdNF-Y* subfamily, and a pair of duplicated genes was determined by a correlation identity of two genes >70% (Figure S2C and Table S3). The results showed four duplicated pairs: *MdNF-YB5/MdNF-YB15* (76.9%), *MdNF-YB8/MdNF-YB18* (94.4%), *MdNF-YC1/MdNF-YC6* (77.1%), and *MdNF-YC2/MdNF-YC8* (87.4%). All four duplicated pairs of genes were located on different chromosomes (Figure S2B), indicating that segmental duplication events might have contributed to the expansion of the *MdNF-Y* gene family in the apple genome.

To further predict the approximate time of the segmental duplication events, we used DnaSP V6.0 software to predict nonsynonymous (Ka) and synonymous (Ks), as well as their ratio Ka/Ks (Table S2). The ratios of the four duplicated pairs were all lower than 1, suggesting the possibility of negative selective pressure associated with the conserved protein sequences. The origin dates of the four pairs of duplicated genes can be traced back from 4.65 to 9.19 million years ago.

### 2.3. NF-Y Members Behave Differently in Different M. sieversii Organs and in Response to Drought Stress

**Figure 2:**
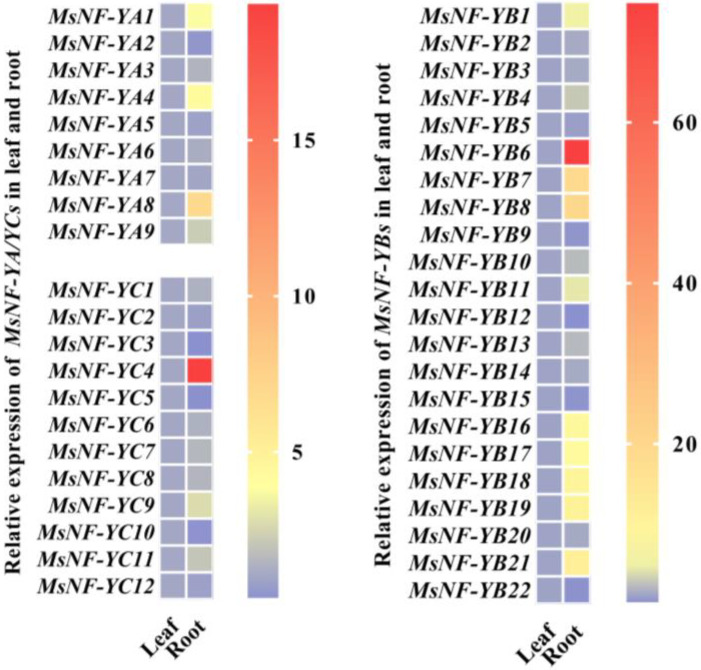
*MsNF-Ys* behaved differently in the leaves and roots. The heat map shows the relative mRNA level of *MsNF-Y* genes in the leaves and roots. Apple *Histone H3* was chosen for the internal quantification control of the *MsNF-Y* genes. The expression of each *MsNF-Y* gene in the leaves was normalized to 1.

*Malus sieversii* Roem. (*M. sieversii*), an ancestral species of the modern apple cultivars, is a type of apple tree with strong drought tolerance [11,39]. To investigate the potential role of each apple NF-Y in the drought response, we conducted an expression analysis of the *NF-Y* family members in different *M. sieversii* organs (Figure 2). All of the *MsNF-Y* transcripts could be detected in the leaves and roots, with diverse expression levels. Strikingly, most of *MsNF-Y* genes showed a higher expression level in the roots than in leaves. For instance, there were 10 *MsNF-Y* genes with a more than five-fold higher root expression level than leaf expression level, especially *MsNF-YB6,* which had a nearly 75-fold higher expression level in the roots than in the leaves (Figure S3). This diversity of *MsNF-Ys* expression indicated the different roles of *MsNF-Ys* in apple growth and development.

**Figure 3:**
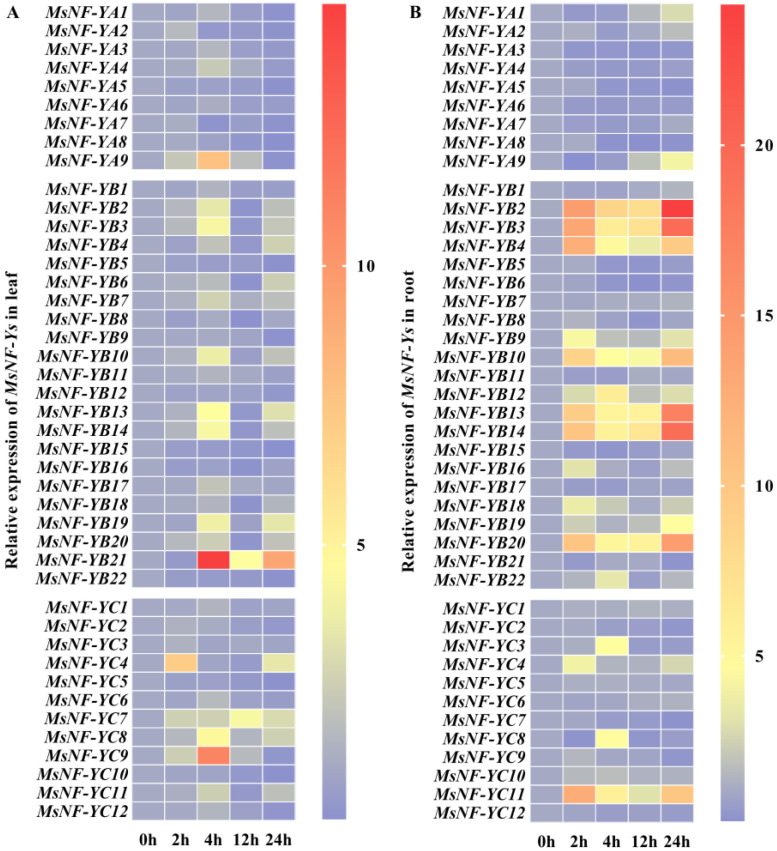
*MsNF-Ys* of the leaves and roots behaved differently in response to drought stress. The heat map shows the relative mRNA level of *MsNF-Y* genes in the leaves and roots under drought treatment, compared to the expression level of *MsNF-Y* genes under 0 h treatment. (**A**) The expression of *MsNF-Y* genes in the leaves under drought treatment. (**B**) The expression of *MsNF-Y* genes in the roots under drought treatment. Apple *Histone H3* was chosen for the internal quantification control of *MsNF-Y* genes. The expression of each *MsNF-Y* gene in the leaves and roots at 0 h treatment was normalized to 1.

Previous studies have shown that the *NF-Y* gene family is closely related to drought tolerance in some species, including *Arabidopsis* and peaches [19,32,33,34]. We used RT-qPCR to identify the drought-responsive *MsNF-Y* genes in *M. sieversii* leaves and roots under drought treatment (Figure 3). Genes with changes in expression level greater than two-fold were selected as candidates (Table S5). In the leaves, a total of 19 genes, including two *MsNF-YAs*, 12 *MsNF-YBs*, and five *MsNF-YCs*, showed upregulated expression levels in response to drought stress. It is worth noting that nine of these genes had more than a four-fold expression change (Figure S4). *MsNF-YA9*, *MsNF-YB3/13/14/21*, and *MsNF-YC7/8/9* were upregulated within 4 h but decreased rapidly as the treatment progressed. Interestingly, the expression patterns of *MsNF-YB21* and *MsNF-YC4* were different than the others. For *MsNF-YC4*, the expression level reached a peak (about seven-fold) within 2 h and then reduced rapidly, before recovering at 24 h after treatment.

As for the expression level of *MsNF-Y* genes in the roots, there were 19 genes displayed incrementally, including two *MsNF-YAs*, 13 *MsNF-YBs*, and four *MsNF-YCs* (Table S5). Among them, 12 out of these 19 genes had a more than four-fold change in expression level (Figure S5). In comparison with the leaves, nine genes (*MsNF-YB2/3/4/10/13/14/19/20* and *MsNF-YC11*) responded quickly to drought stress within 2 h, which is earlier than in the leaves. In particular, *MsNF-YB12* and *MsNF-YC3/8* reached their peak expression level at 4 h, which was then reduced to the normal level by 24 h. The diverse expression patterns of *MsNF-Y* genes in the leaves and roots under drought stress suggested that NF-Y members might contribute to the spatiotemporal characteristics of the apple drought response.

### 2.4. Cis-Elements in the Promoters of MdNF-Y Genes

Genes with similar expression patterns are likely to share common regulatory *cis*-elements in their promoters. Hence, to analyze the regulatory properties of *MdNF-Y* in response to abiotic stress, we selected 12 reported *cis*-elements that are involved in the stress response and analyzed their present situation in the promoters of 43 *MdNF-Y* genes (Figure S6 and Table S4). Of the 12 *cis*-elements, five elements were linked with the drought response, including ABREs, MBSs, G-Boxes, W-Boxes, and DREs. Strikingly, at least two drought-responsive *cis*-elements were observed on each *MdNF-Y* promoter (*proMdNF-Ys*), consistent with the finding that a large number of apple *NF-Y* genes are drought-responsive. Notably, the numbers of drought-responsive *cis*-elements in the 43 *proMdNF-Ys* ranged from two (*proMdNF-YA3/YC2/YC5/YC8*) to 21 (*proMdNF-YC4*). Moreover, other stress-related and hormone-related *cis*-elements, other than the drought response, were analyzed in *proMdNF-Ys*. The results showed that 33 LTR (low temperature responsive), 31 WUN (wound responsive), six GARE and 18 P-Box (gibberellin responsive), 50 CGTCA motif and 41 TGACG motif (jasmonic acid responsive), and 30 TCA-elements (salicylic acid responsive) were found on the promoter of *MdNF-Ys*. The total number of these *cis*-elements ranged from zero (*proMdNF-YB6*) to 11 (*proMdNF-YC3*). Taken together, the *cis*-element analysis in the *MdNF-Y* promoters suggested that apple *NF-Ys* respond to various environmental stresses.

### 2.5. MsNF-YB21 Overexpression Improves Osmotic Stress Resistance in Arabidopsis

**Figure 4:**
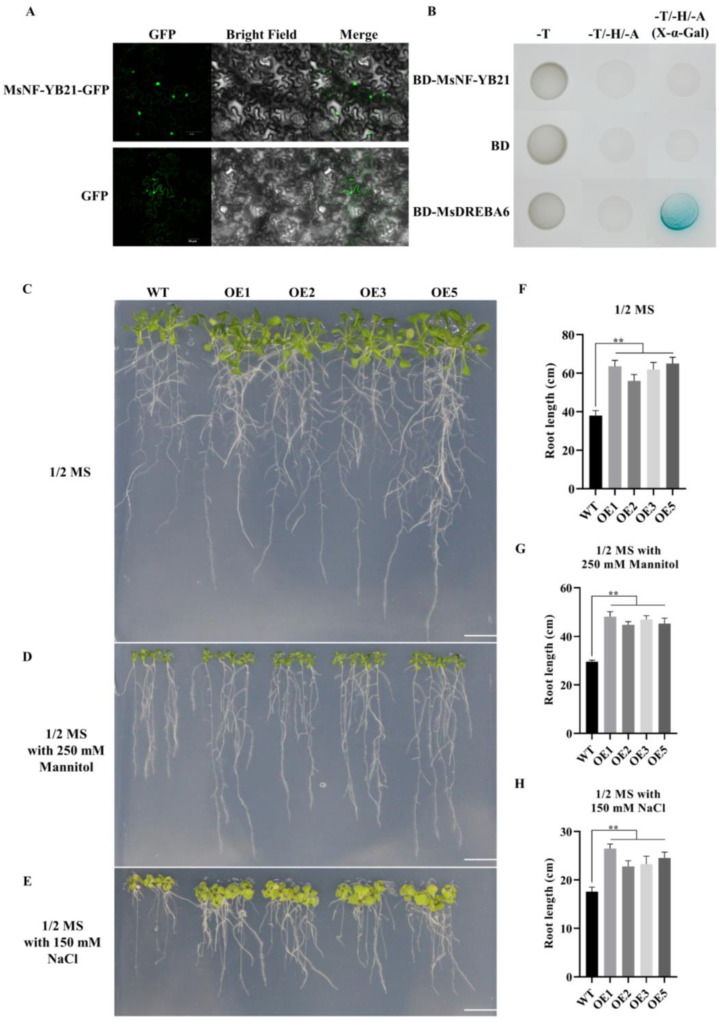
Characteristics of MsNF-YB21 and plant growth features of *MsNF-YB21* overexpression lines under normal and stress conditions. (**A**) Subcellular localization of transiently expressed *35S::GFP* and *35S::MdNF-YB21::GFP* in *N. benthamiana* leaves. The GFP (green fluorescent protein) signal was observed by fluorescence microscopy 48 h later. Scale bars = 50 μm. (**B**) Transcription activation activity of MsNF-YB21 in yeast cells. MsDREBA6 and BD (binding domain) were used as positive and negative controls, respectively. Seven day old WT (wild type) and OE (*MsNF-YB21* overexpression) seedlings were transferred to 1/2 Murashige and Skoog (MS) medium, supplemented with 250 mM mannitol (**D**) or 150 mM NaCl (**E**), while standard 1/2 MS was regarded as the control (**C**). Scale bars = 1.0 cm. The primary root length of all lines under different growth conditions was recorded in (**F**–**H**). Values are the means ± SE (standard error) from three biological replicates. Significant differences of each root length were assessed using Student’s *t*-test in comparison to WT. (Double asterisks denote *p* < 0.01).

As *MsNF-YB21* is dramatically induced by drought treatment (Figure 3 and Figure S4), we selected *MsNF-YB21* to further explore its role in the drought response. The subcellular localization assay showed that the *MsNF-YB21*-GFP fusion protein was localized to the nucleus and cell membrane in tobacco leaves (Figure 4A). Furthermore, we performed a transcriptional activation activity assay in yeast and found that MsNF-YB21-GAL4 BD was unable to induce the reporter gene compared with the positive control (MsDREB6.2-GAL4 BD), indicating that MsNF-YB21 might not have transcriptional activation activity (Figure 4B).

To investigate the possible biological functions of *MsNF-YB21* in the regulation of plant growth and resistance to osmotic stresses, we generated transgenic *Arabidopsis* homozygous lines overexpressing *MsNF-YB21* under the control of the CaMV35S promoter. After verification of the transcript level using RT-qPCR, four transgenic lines (OE1, OE2, OE3, and OE5) were selected for further analysis (Supplementary Figure S7). 

To understand the function of *MsNF-YB21*, seven-day old WT (wild type, *Col-0*) and transgenic *Arabidopsis* seeds were either kept in 1/2 MS medium or transferred onto the 1/2 MS medium with 250 mM mannitol or 150 mM NaCl for 14 days. Compared to WT, all the OE (*MsNF-YB21* overexpression) lines showed larger shoots and longer roots under normal conditions (Figure 4C). The average primary root length of the OE lines ranged from 56.0 to 65.1 cm, which was significantly longer than the 38.0 cm of the WT (Figure 4F). Importantly, after 250 mM mannitol or 150 mM NaCl treatment, the growth of all the *Arabidopsis* seedlings was obviously inhibited, while the primary root length of the OE lines was still remarkably longer than the WT (Figure 4D,E,G,H). All these results indicated that *MsNF-YB21* positively regulates plant growth and osmotic stress tolerance.

### 2.6. MsNF-YB21 Alleviates Osmotic-Induced Oxidative Stress by Improving the Antioxidant Capacity

**Figure 5:**
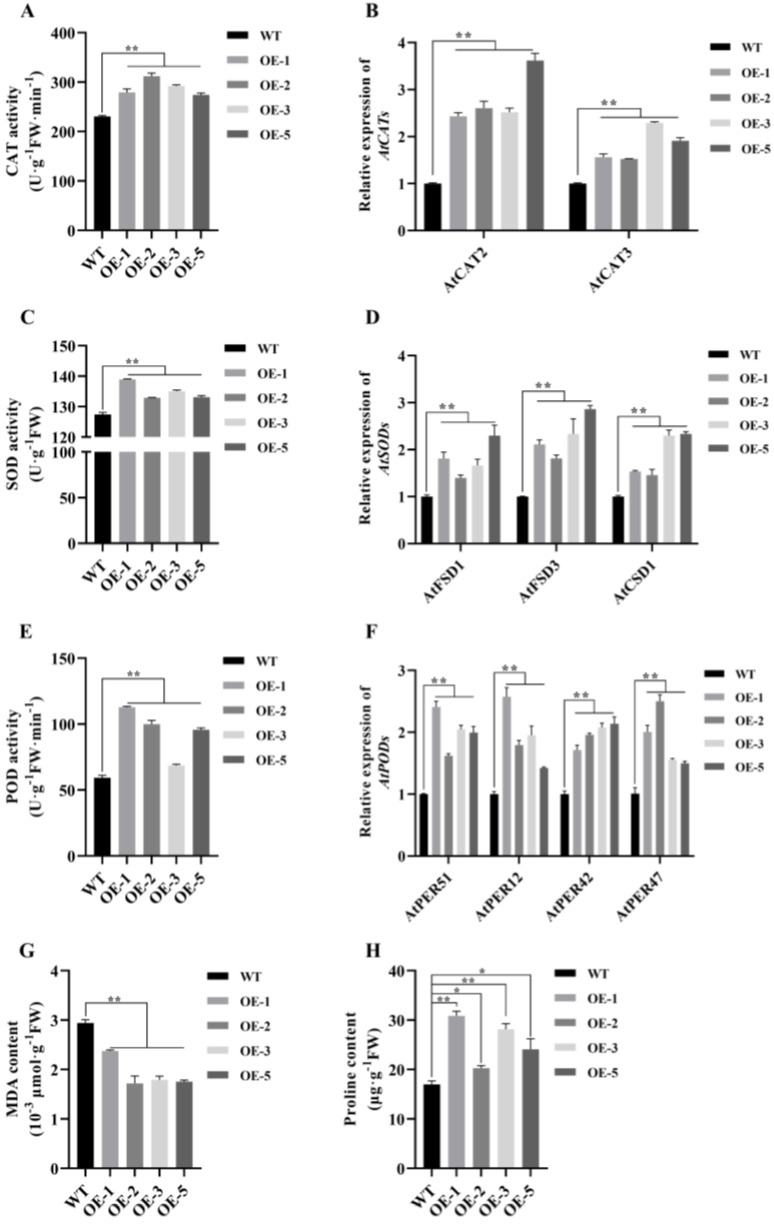
Antioxidant enzyme activity, compatible solute concentration, and expression level of the genes encoding *Arabidopsis* catalase (CAT), superoxide dismutase (SOD), and peroxidase (POD) in *MsNF-YB21* overexpression lines were higher than in WT under normal conditions. (**A**) CAT activity analysis. (**B**) SOD activity analysis. (**C**) POD activity analysis. (**D**) Expression patterns of the CAT genes. (**E**) Expression patterns of the SOD genes. (**F**) Expression patterns of the POD genes. (**G**) Malondialdehyde (MDA) content analysis. (**H**) Proline content analysis. *AtACT2* was chosen for the internal quantification control of *Arabidopsis* genes. Data are shown as means ± SE from three replicates of each 50 representative plants used. Significant differences were assessed using Student’s *t*-test in comparison to WT (single asterisks denote *p* < 0.05; double asterisks denote *p* < 0.01).

**Figure 6:**
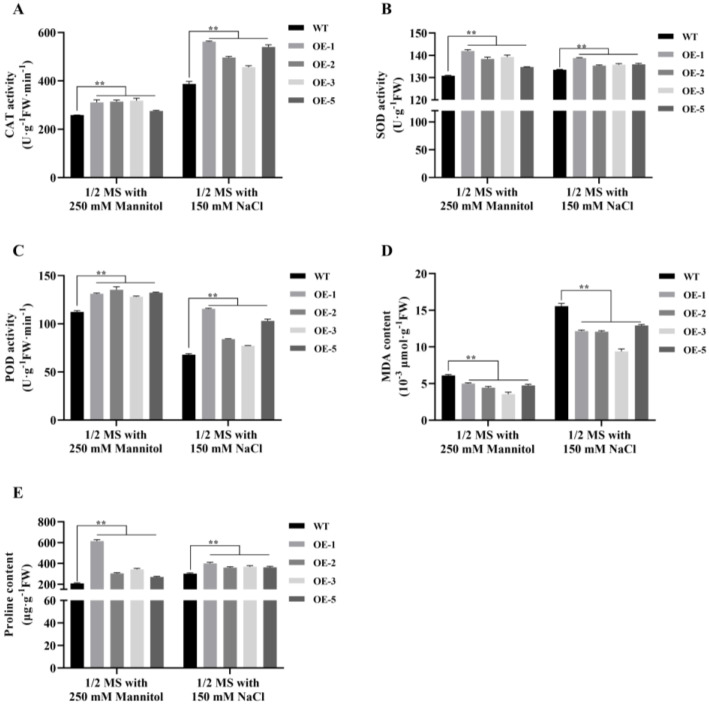
Antioxidant enzyme activity and the compatible solute concentration of *MsNF-YB21* overexpression lines were obviously improved compared with WT under stress conditions. (**A**) CAT activity analysis. (**B**) SOD activity analysis. (**C**) POD activity analysis. (**D**) MDA content analysis. (**E**) Proline content analysis. Data are shown as the means ± SE from three replicates in each 50 representative plants used. Significant differences were assessed using Student’s *t*-test in comparison to WT (double asterisks denote *p* < 0.01).

Antioxidant enzymes (AEs) have important roles during osmotic stresses. To determine the antioxidant ability, catalase (CAT), superoxide dismutase (SOD), and peroxidase (POD) activity were measured in *MsNF-YB21* overexpression lines. Consistently with the physiological characteristics (Figure 4A), all the OE lines were shown to harbor significantly higher AE activity than the WT plants under normal conditions (Figure 5A,C,E). In addition, the expression level of the CAT, SOD, and POD genes, which encoded the AEs, was shown to be increased in *MsNF-YB21* OE plants (Figure 5B,D,F), indicating that these genes can be regulated by MsNF-YB21. Similarly, the *MsNF-YB21* OE plants also displayed higher AE activity than WT upon mannitol or NaCl treatment (Figure 6A–C), indicating that MsNF-YB21 might alleviate osmotic-induced oxidative stress by improving the antioxidant capacity. All these results suggested that MsNF-YB21 controls AE activity by regulating the AE-encoding genes.

Malondialdehyde (MDA) is one of the products derived from plasma membrane peroxidation, which can reflect the degree of cell damage. To determine whether the enhanced drought tolerance of *MsNF-YB21* OE plants is connected with reduced cell damage, we measured the MDA content in WT and *MsNF-YB21* OE plants under normal and stress treatments. The results showed that OE lines had obviously lower MDA than that in WT plants (Figure 5G and Figure 6D). Proline is an important osmotic protective substance in response to abiotic stress, which can protect plants from damage caused by osmotic stresses to a certain degree. The proline content of the overexpression lines was higher than that in the WT plants under normal and osmotic stresses conditions (Figure 5H and Figure 6E). Taken together, these results suggested that MsNF-YB21 might act to reduce cellular membrane injury and maintain cellular stability by reducing and promoting MDA and proline accumulation, respectively, thus avoiding excessive damage from osmotic stresses.

### 2.7. MsNF-YB21 Can Interact with MsNF-YC8

**Figure 7:**
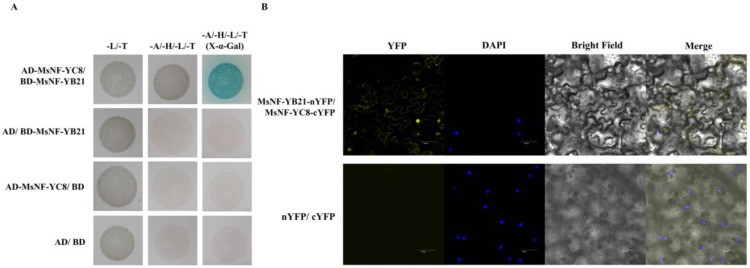
MsNF-YB21 interacts with MsNF-YC8. (**A**) Y2H assays of the interaction between AD-MsNF-YC8 and BD-MsNF-YB21. AD, activation domain. BD, binding domain. (**B**) BiFC assay showing that MsNF-YB21 interacted with MsNF-YC8 in the nucleus and membrane of *N. benthamiana* epidermal cells. The nuclear dye DAPI (blue) was applied to visualize the nucleus. Scale bars = 50 μm.

Previous studies have reported that a dimer of NF-YBs and NF-YCs is formed before facilitating the NF-Y trimer complex with NF-YA subunits, given that the expression pattern of *MsNF-YC8* was similar to *MsNF-YB21* in the leaves (Figure 3A and Figure S4). In order to examine whether MsNF-YB21 could interact with MsNF-YC8, we conducted Y2H and BiFC assays. As shown in Figure 7A, yeast cells transformed with MsNF-YB21 and MsNF-YC8 proteins were able to grow on an SD-A-H-L-T plate, suggesting an interaction between MsNF-YB21 and MsNF-YC8 in yeast. Furthermore, the MsNF-YB21 and MsNF-YC8 interaction was confirmed using BiFC assay. The results showed that the YFP signal was observed in the nuclei and cell membrane of *Nicotiana benthamiana* epidermal cells co-expressing MsNF-YB21 and MsNF-YC8 (Figure 7B). These observations indicated that MsNF-YB21 interacts with MsNF-YC8 in the nucleus and cell membrane in the plant cell.

## 3. Discussion

### 3.1. The MdNF-Ys Include 43 Members

Previous evidence has proven that each NF-Y clade is encoded by only one member in humans and yeast, while various numbers of NF-Y members for each NF-Y subunit have been identified in plants [40]. In recent decades, researchers have explored the important function of NF-Ys in plants. However, the function of most NF-Y proteins in apple remains poorly understood. In this study, a total of 43 NF-Y members, divided into three clades (MdNF-YA, MdNF-YB, and MdNF-YC), were identified in apples, more than in *Arabidopsis* (36 AtNF-Y members) and peaches (24 PpNF-Y members), but less than in *Glycine max* (59 GmNF-Y members) [17,18,19]. We speculate that these differences in gene numbers might be linked to variations in genome size, as apples and soybeans have larger genomes. Strikingly, the number of *NF-YA* and *NF-YC* genes was different from the number found by Qu et al. [38]. For *NF-YA*, two candidate genes (MD02G1309800 and MD12G1042100) identified in Qu et al. [38] did not have a complete NF-YA domain in our analysis; thus, we removed these two genes. For *NF-YC*, we added two more candidate genes (MD02G1273400 and MD07G1042300) because of E-values less than 10^−10^.

Phylogenetic analysis revealed that 43 MdNF-Y members could be divided into three subunits: MdNF-YA, MdNF-YB, and MdNF-YC. The phylogenetic tree was shaped similarly to those reported in previous studies, including peaches, tea, and watermelon [19,40,41], implying that most of the *NF-Y* genes were evolutionarily conserved. Surprisingly, MdNF-Y proteins were closer to the PpNF-Ys rather than to *Arabidopsis* NF-Y proteins (Figure 1).

Gene families can be enlarged by duplication events, which may lead to functional diversity. In our work, we found four paralogous *NF-Y* genes located on different chromosomes (Figure S2B), implying that segmental duplication events contributed to the expansion of *MdNF-Ys*. The Ka/Ks ratios of the four paralogous *MdNF-Ys* were less than 1, indicating the corresponding paralogous MdNF-Y proteins were conserved. The oldest date of the duplication events among the four pairs of paralogous MdNF-Ys could be 9.19 million years ago, suggesting that this might be an ancient gene family (Table S2).

### 3.2. Diverse Expression Patterns of MsNF-Ys in Different Tissues and under Drought Stress

The expression analysis showed that most *MsNF-Y* genes are highly expressed in the roots, such as *MsNF-YA8*, *MsNF-YB6*, and *MsNF-YC4*, indicating that they might be involved in root growth. It has been shown that increased expression of *AtNF-YA2* and *AtNF-YA10* (which are influenced by knockdown of the expression of microRNA169) could lead to enhanced primary root elongation [42]. Our phylogenetic analysis (Figure S1) showed that *MsNF-YA8* clustered with *AtNF-YA2* and *AtNF-YA10*, indicating that this gene might also have a role in root development. Some *MsNF-Ys*, such as *MsNF-YC12*, were expressed in both the leaves and the roots, showing that they might have multiple roles in the developmental process. In addition, the remaining *MsNF-Ys*, such as *MsNF-YB15* and *MsNF-YC5,* were specifically expressed in the leaves, implying that they might participate in leaf morphogenesis. The expression patterns of *MsNF-Ys* in different organs could provide useful information for a further understanding of the biological functions of *MsNF-Ys*.

Increasing evidence indicates that NF-Ys play essential roles in resistance to drought stress in multiple plant species [19,43]. *M. sieversii* is widely used as a rootstock to improve the drought resistance of apple scions [11,39,44], but the potential mechanisms remain unclear. Our *cis*-element analysis showed that at least two drought-responsive *cis*-elements were found in each *MsNF-Y* promoter sequence (Figure S6 and Table S4), suggesting that *MsNF-Y* genes might participate in the drought-responsive pathway. Moreover, the drought stress treatment in *M. sieversii* leaves and roots showed that 19 different *MsNF-Ys* are able to respond to drought stress, indicating the involvement of these genes in the drought stress response. Therefore, further studies are required to identify the functions of *MsNF-Ys* in drought stress. Previous studies have revealed that overexpression of *AtNF-YA2*/*3*/*5*/*7*/*10* could improve the tolerance of transgenic plants to drought stress, and drought treatment can induce the expression of *PpNF-YA3*/*5*/*6* [19]. *MsNF-YA1/9*, which were clustered with those genes, were induced by drought treatment in the roots and leaves (Figure S4 and Table S5). Moreover, three ABRE, one MBS, and four G-Boxes were observed in the *MsNF-YA9* promoter (Table S4), indicating that the *MsNF-YA9* gene might play important roles in the drought response via an ABA-dependent pathway. In addition, compared with the control, *MsNF-YB21* was upregulated nearly 15-fold at 4 h under drought treatment in the leaves, and *cis*-element analysis of *MsNF-YB21* showed that two ABRE, one G-Box, and one W-Box were located in the promoter sequence, suggesting this gene could be an important candidate for the drought response. Furthermore, *AtNF-YC9* is considered to improve the resistance of drought stress in *Arabidopsis*, and *PpNF-YC4* was increased by drought treatment. *MsNF-YC8* clustered with *AtNF-YC9* and *PpNF-YC4* in subgroup II and was significantly upregulated at 4 h after drought treatment in both the leaves and roots, while *MsNF-YC3* and *MsNF-YC9* were highly expressed in the roots and leaves, respectively. *Cis*-element analysis showed that all three genes contained drought-responsive elements in their promoter, indicative of their involvement in the process of drought response. Thus, future studies may focus on the functions of different *MsNF-Y* genes, to better understand the mechanism of drought tolerance in *M. sieversii*.

### 3.3. MsNF-YB21 Regulates the Plant Growth and Osmotic Stress Response

Phylogenetic analysis showed that MsNF-YB21 clustered with AtNF-YB2 and AtNF-YB3 (Figure S8), which have been reported to participate in regulating plant growth and the response to abiotic stress [26,43]. Overexpression of garlic *AsNF-YB3* in tobacco, an orthologous gene of *AtNF-YB3*, enhances both root and shoot growth [45]. Similarly, other research found that overexpression of potato *StNF-YB3.1* led to faster growth rates and greater plant heights at an early stage [46]. In addition, *ZmNF-YB16* and *TaNF-YB3;l*, orthologous of *AtNF-YB3*, mediate the drought adaptation of maize and wheat, respectively [35,47]. Our study found that heterologous expression of *MsNF-YB21* in *Arabidopsis* promotes the growth of shoots and the elongation of roots in transgenic plants under osmotic stresses (Figure 4D,E). In contrast to the wild type, reduced cellular damage concomitant with enhanced physiological characteristics related to osmotic resistance was observed in *MsNF-YB21* overexpression lines (Figure 6D,E).

Antioxidant systems are essential in plants responding to osmotic stresses. Antioxidant enzymes (i.e., CAT, SOD, and POD) and compatible solutes (i.e., proline) play important roles in plant resistance to osmotic stresses. It has been shown that overexpression of *TaNF-YB3;l* contributed to improved enzymatic SOD, CAT, and POD activities, as well as elevated osmolyte content, under drought stress [47]. In addition, overexpression of *ZmNF-YB16* in maize substantially increased the antioxidant capacity, and 10 genes encoding peroxidase were upregulated in overexpression lines after drought stress [35]. These studies suggest that *NF-YB* genes improve plant resistance to stress by regulating antioxidant gene expression and antioxidant activity. Our work revealed that the *MsNF-YB21* overexpression lines also exhibited enhanced CAT, SOD, and POD activities and elevated proline content under normal conditions and osmotic stresses (Figure 5A,C,E and Figure 6A–C). Furthermore, two CAT genes, three SOD genes, and four POD genes were upregulated in *MsNF-YB21* OE lines (Figure 5B,D,F). These results hint that MsNF-YB21 improved the enzyme activities in OE lines through the regulation of AEs encoding genes expression, increasing the osmotic stress resistance of transgenic plants.

### 3.4. MsNF-YC8 Could Serve as a Potential Partner of MsNF-YB21 in Response to Stresses in Apples

Our transgenic assay showed that MsNF-YB21 plays an important role in osmotic tolerance, and expression of genes related to osmotic stress is enhanced, indicating that MsNF-YB21 might be able to activate transcription of downstream genes. However, despite the fact that MsNF-YB21 is localized in the nucleus, our transcriptional activity assay in yeast revealed a lack of such activity. Thus, the results suggested that other factors related to transcriptional activity are needed to facilitate the action of MsNF-YB21. Therefore, it will be interesting to explore the proteins that interact with MsNF-YB21, to confer its functions in osmotic stress.

Previous studies have proven that NF-YB and NF-YC can form a dimer before interacting with NF-YA or other proteins. For instance, *Arabidopsis* NF-YC3/4 and 9 can physically interact with NF-YB2/3 and CO, and then influence the flowering time [26]. *Picea wilsonii* PwNF-YB3, an orthologous protein of AtNF-YB3, shows a lack of transcriptional activation properties [28]. Researchers have proven that PwNF-YB3 can interact with PwHAP5, and heterologous expression of this gene in *Arabidopsis* can induce the expression of drought response genes, indicating that PwNF-YB3 can activate the expression of downstream genes [28]. Another study showed that, despite the absence of transcriptional activity, PdNF-YB21 is able to activate *PdNCED3* gene expression through the interaction with PdFUS3, thus regulating the growth of the poplar root system [48]. These results hint that NF-YB21 does not act alone and may recruit other TFs or proteins to activate the expression of downstream genes.

In this study, we found that *MsNF-YC8* shares a similar expression pattern with *MsNF-YB21* in the leaves (Figure 3 and Figure S4). Further results revealed that these two subunits can interact in yeast and plants; thus, MsNF-YB21 and MsNF-YC8 may be involved in a functional dimeric complex in response to drought stress in apples.

## 4. Materials and Methods

### 4.1. Identification of NF-Y Transcription Factor Family Members in Apples

In order to identity NF-Y TF family members in the apple genome, 36 *Arabidopsis* NF-Y protein sequences were downloaded from the TAIR database (http://www.arabidopsis.org/ (accessed date on 5 December 2018)) and used as query sequences. The protein-to-protein BLAST (blastp) program (https://www.rosaceae.org/blast/protein/protein (accessed date on 5 December 2018)) was applied with an E-value cutoff of 10^−10^ to identify potential NF-Y TFs in the apple genome (GDDH13 v1.1) database (https://www.rosaceae.org/species/malus/malus_x_domestica/genome_GDDH13_v1.1 (accessed date on 5 December 2018)). In addition, the hidden Markov model (HMM) profile of the NF-Y domain (PF02045 and PF00808) from the Pfam database (http://pfam.xfam.org/ (accessed date on 5 December 2018)) was also used as a query to identify all the NF-Y domain-containing sequences in the apple genome database with the BLAST-p searching program [49]. The sequences of the two methods were merged and then analyzed by a similarity sequence comparison to remove the redundant NF-Ys. The results of the remaining NF-Ys were further confirmed by the CCD (https://www.ncbi.nlm.nih.gov/cdd/ (accessed date on 5 December 2018)) and SMARAT (http://smart.embl-heidelberg.de/ (accessed date on 5 December 2018)) programs, to verify the existence of the complete NF-Y domains. After all of the analysis, the remaining sequences were considered to be MdNF-Ys. The identified MdNF-Ys were named according to their chromosomal position.

### 4.2. Bioinformatics Analysis of MdNF-Y Genes

The IPC online software (http://isoelectric.org/ (accessed date on 5 December 2018)) was used to predict the molecular weights and isoelectric points of the MdNF-Y proteins. Subcellular localization was predicted using the Plant-mPLoc program on the Cell-PLoc 2.0 website (http://www.csbio.sjtu.edu.cn/bioinf/Cell-PLoc-2/ (accessed date on 5 December 2018)). The hydrophilicity prediction of MdNF-Y proteins was calculated through the GRAVY CALCULATOR online program (http://www.gravy-calculator.de/ (accessed date on 5 December 2018)). The map of the distribution of *MdNF-Y* genes on the apple genome was drawn using the MapINspect tool (available online: http://mapinspect.software.informer.com (accessed date on 5 December 2018)) and modified manually with annotation. The alignment of MdNF-Y protein sequences was carried out using Clustal X software. Mega X software [50] (http://www.megasoftware.net/ (accessed date on 5 December 2018)) was used for constructing the phylogenetic trees of NF-Y proteins (*Malus × domestica*, *Arabidopsis*, and *Prunus persica*) by the neighbor-joining (NJ) method, with 1000 bootstrap replicates. The NF-Y proteins of *Arabidopsis* and *Prunus persica* [19] were downloaded from TAIR (http://www.arabidopsis.org/ (accessed date on 5 December 2018)) and the peach protein database (version 2.0, https://www.rosaceae.org/blast/protein/protein (accessed date on 5 December 2018)). The gene duplication event analysis of the *MdNF-Ys* was calculated on the basis of the identity of their CDSs. The percentage identity matrix of the *MdNF-Ys* was analyzed in BioEdit software and visualized with the TBtools toolkit [51]. A pair of duplicated *MdNF-Ys* was defined when they shared >70% identity at the nucleotide level. The parameters Ks (synonymous substitution rate) and Ka (nonsynonymous substitution rate) were calculated using the program DNASP 6.0. The Ka/Ks ratio between paralogs was analyzed to predict the mode of selection. The approximate time of the duplication events was estimated as in previous studies [19].

Furthermore, the approximate upstream 2000 bp of *MdNF-Y* sequences from the start codon (ATG) were extracted by the TBtools toolkit and regarded as promoter regions. The stress-responsive *cis*-acting elements were analyzed and plotted using the PlantCARE online program (http://bioinformatics.psb.ugent.be/webtools/plantcare/html/ (accessed date on 5 December 2018)) and TBtools toolkit, respectively.

### 4.3. Plant Materials, Growth Conditions, and Drought Stress Treatment

Micropropagated and rooted *M. sieversii* plants were grown in 1/2 Hoagland nutrient solution for 15 days, and then transferred to full-strength Hoagland nutrient solution for normal growth. *Arabidopsis thaliana* ecotype *Columbia-0* (*Col-0*) was used as the wild type (WT) and background for the transformation of *Arabidopsis*. All the plants were grown at 22 °C, with 60% relative humidity, under a 16 h/8 h (day/night) photoperiod. When the plants reached a height of approximately 25 cm, they were used in the drought stress experiments. For the drought stress treatment, three plants with three biological replicates were transferred to a 20% (*w*/*v*) PEG-6000 solution. The leaves and roots were collected separately at 0, 2, 4, 12, and 24 h after treatment. All samples were frozen in liquid nitrogen and stored at −80 °C.

### 4.4. RNA Extraction and Real-Time Quantitative PCR

The total RNA of *M. sieversii* and *Arabidopsis* was extracted, respectively, using the Quick RNA Isolation Kit (CWBIO, Beijing, China) and TRIzol Reagent (CWBIO, Beijing, China). In order to synthesize cDNA, the RNA samples were used as templates with the M-MLV Reverse Transcriptase Kit (Takara Bio, Shiga, Japan). RT-qPCR was performed in a 20 μL reaction system containing 10 μL of 2 × UltraSYBR Mixture (CWBIO) and 0.4 μM of forward and reverse primers, to assess the expression of target genes. The reactions were incubated in a Rotor-Gene Q Machine (Qiagen) for 10 min at 95 °C, followed by 40 cycles of 15 s at 95 °C and 60 s at 60 °C. *AtACT2* and *Histone H3* were chosen for the internal quantification controls of the *Arabidopsis* and apple genes, respectively. Relative expression levels were measured using the 2^−ΔΔCt^ method [52]. The primers are listed in Supplementary Table S6.

### 4.5. Plasmid Construction and Genic Transformation in Arabidopsis

The full-length coding sequences (CDS) of *MsNF-YB21* and *MsNF-YC8* were obtained by the RT-PCR method and cloned into the pTOPO-Blunt Simple Vector (Aidlab, Beijing, China). The full-length CDS of *MsNF-YB21,* lacking the termination codon, was cloned and inserted into the pCAMBIA1302 vector containing a GFP tag to generate an *MsNF-YB21* overexpression vector. All these constructs were introduced into *Agrobacterium tumefaciens* strain EHA105. The primers used are listed in Supplementary Table S7.

As for the transformation of *Arabidopsis*, *Col-0* sterilized seeds were chilled for 4 days at 4 °C, and then germinated on 1/2 Murashige and Skoog (MS) medium with 0.7% (*w*/*v*) agar and 3% (*w*/*v*) sucrose. After 10 days, all the plants were transferred into containers of nutrient-rich potting medium mixed with vermiculite (1:1). The pCAMBIA1302-*MsNF-YB21* plasmid was transformed into *Col-0* plants as previously described [53]. Positive transgenic lines were selected on Murashige and Skoog medium based on hygromycin (50 mg/L) resistance. The expression levels of *MsNF-YB21* in the transgenic plants were determined by RT-qPCR. Finally, four independent homozygous T3 transgenic lines with higher expression (#1, #2, #3, #5) were selected for further investigation.

### 4.6. Seedling Stress Tolerance Analysis

To determine the response of *Arabidopsis* to environmental stress, seeds of WT and homozygous transgenic plants expressing *MsNF-YB21* (OE) were grown in standard 1/2 MS mediums. After 7 days, all of the seedlings were transferred to 1/2 MS medium supplemented with mannitol (250 mM) or NaCl (150 mM), while standard 1/2 MS was regarded as the control. After 14 days of stress treatment, the root lengths of WT and OE plants in each treatment were recorded. Fifty seedlings of each line were analyzed, with three biological replicates.

### 4.7. Subcellular Localization and Transcriptional Activity Analysis of MsNF-YB21

To examine the subcellular localization of *MsNF-YB21*, we used the *MsNF-YB21* overexpression vector described as above. The constructs were delivered into epidermal cells of approximately 4 week old *Nicotiana benthamiana* (*N. benthamiana*) leaves. After 2 days of incubation, the fluorescence signal was detected using an Olympus laser-scanning confocal microscope.

For transcriptional activity analysis, the full-length CDS of *MsNF-YB21* was fused to the pGBKT7 vector containing the GAL4 DNA binding domain (BD). The recombination plasmid was transformed into yeast strain AH109. The transformants were screened and *MsDREB6.2*-GAL4 BD was used as the positive control.

### 4.8. Analysis of Interactions between MsNF-YB21 and MsNF-YC8 by Y2H and BiFC Assay

For the yeast two-hybrid assay (Y2H), the full-length CDS of *MsNF-YC8* was fused to the pGATD7 vector containing the GAL4 activation domain (AD). The double transformation was carried out by introducing *MsNF-YC8-*GAL4 AD and *MsNF-YB21-*GAL4 BD into the yeast strain AH109. The transformants were screened.

For bimolecular fluorescence complementation assay (BiFC), the *pSPYNE-35S* vector containing an N-YFP tag and *pSPYCE-35S* vector containing a C-YFP tag were used for cloning [54]. The full-length CDSs of *MsNF-YB21* and *MsNF-YC8* were separately fused in frame with N-YFP and C-YFP. The recombination plasmids were introduced into *Agrobacterium tumefaciens* strain EHA105. The constructs were delivered into epidermal cells of approximately 4 week old *N. benthamiana* leaves. After 2 days of incubation, the fluorescence signal was detected using an Olympus laser-scanning confocal microscope.

### 4.9. Extraction and Assay of Antioxidant Enzymes

Treated or untreated seedlings (0.5 g) were extracted and homogenized in 5 mL of 3% (*w*/*v*) sulfosalicylic acid. After heating for 10 min at 100 °C, the mixture was cooled quickly in an ice bath. A 2 mL aliquot of the supernatant was mixed with 3 mL of 2.5% acid ninhydrin and 2 mL of glacial acetic acid, and this mixture was boiled at 100 °C for 40 min in a water bath, and then terminated on ice, before the proline content was determined as described [55]. 

Treated or untreated seedlings (0.5 g) were homogenized in 0.3% TBA and 10% trichloroacetic acid. After heating for 10 min at 100 °C, the mixture was cooled quickly in an ice bath. The mixture was centrifuged at 12,000× *g* for 10 min, and the resulting supernatant was used for determining the malondialdehyde (MDA) concentrations. The MDA concentrations were determined as described [56].

Treated or untreated seedlings (0.5 g) were ground to a fine powder under liquid nitrogen and suspended in cold extraction buffer (50 mM potassium phosphate buffer, pH 7.8, 3% (*w*/*v*) polyvinylpolypyrrolidone). After centrifugation at 12,000× *g* for 10 min at 4 °C, the supernatant was transferred to a new tube for further use. The activity of catalase (CAT), superoxide dismutase (SOD), and peroxidase (POD) was measured as described previously [57].

### 4.10. Accession Numbers

The accession numbers are shown in Table S8.

## 5. Conclusions

In this study, we systematically investigated apple *NF-Y* genes and conducted initial functional characterization of MdNF-YB21 in the drought response. A total of 43 *MdNF-Y* genes were identified in the apple genome, including nine *MdNF-YAs*, 22 *MdNF-YBs*, and 12 *MdNF-YCs*. Their major characteristics, including phylogenetic relationships, genomic distributions, duplication events, and stress-responsive *cis*-elements in the promoters, were systematically analyzed. RT-qPCR analysis revealed that the *NF-Y* members displayed differential expression patterns in *M. sieversii* roots and leaves. In addition, many *MsNF-Y* genes were induced upon drought stress; for instance, *MsNF-YB21* showed prominent upregulation in response to drought. Overexpression of *MsNF-YB21* in *Arabidopsis* conferred improved osmotic tolerance through regulating the antioxidant systems, including antioxidant enzymes and compatible solutes. Genes encoding catalase (*AtCAT2*, *AtCAT3*), superoxide dismutase (*AtFSD1*, *AtFSD3*, *AtCSD1*), and peroxidase (*AtPER12*, *AtPER42*, *AtPER47*, *AtPER51*) exhibited upregulated expression in *MsNF-YB21* overexpression lines. These results may offer a useful foundation for functional studies of MsNF-YB21, and they support the potential application of MsNF-YB21 in the improvement of apple abiotic stress tolerance via biotechnological strategies.

## Data Availability

The *Arabidopsis* and *Prunus persica* NF-Y protein sequences were downloaded from the *Arabidopsis* information source (TAIR) database (http://www.arabidopsis.org), and the peach protein database (version 2.0, https://www.rosaceae.org/blast/protein/protein).

## Supplementary Materials

The following are available online at https://www.mdpi.com/article/10.3390/ijms22189777/s1, Figure S1: The phylogenetic trees of MdNF-Ys, AtNF-Ys, and PpNF-Ys proteins constructed by MEGA7 with NJ method and a bootstrap of 1000 replicates. (A) NF-YA subfamily. (B) NF-YB subfamily. (C) NF-YB subfamily, Figure S2: Genomic distributions of NF-Y transcription factors on apple chromosomes and nucleotide-level identity matrix analysis. (A) The counted number of *MdNF-Ys* distributed on each chromosome. (B) The distribution and duplication events of 43 *MdNF-Ys* in apple genome. Four pairs of *MdNF-Y* paralogous genes were connected with red lines. (C) The nucleotide-level identity matrix analysis of *MdNF-Ys*. The pairs of duplicated genes were determined by the correlation identity of two genes >70% and colored in red, Figure S3: Expression level of *MsNF-Y* members greater than five-fold in root than in leaf. The expression of each *MsNF-Y* genes in leaf were normalized to 1. Values are the means ± SE from three biological replicates. Significant differences of each gene were assessed using Student’s *t*-test in comparison to leaf. (Double asterisks denote *p* < 0.01), Figure S4: Fold change of *MsNF-Y* members expression level exceeds 4 at 2, 4, 12, and 24 h after drought treatment in leaf. The expression of each *MsNF-Y* gene in leaf under 0 h treatment was normalized to 1. Values are the means ± SE from three biological replicates. Significant differences of each gene assessed using one-way ANOVA. (Lowercase letters denote *p* < 0.05), Figure S5: Fold change of *MsNF-Y* members expression level exceeds 4 at 2, 4, 12, and 24 h after drought treatment in root. The expression of each *MsNF-Y* genes in root under 0 h treatment was normalized to 1. Values are the means ± SE from three biological replicates. Significant differences of each gene assessed using one-way ANOVA. (Lowercase letters denote *p* < 0.05), Figure S6: Schematic model of stress-responsive *cis*-elements in the promoter sequences of *MdNF-Y* genes. MBS represents MYB binding site; ABRE represents abscisic acid (ABA)-responsive element; DRE represents dehydration-responsive element; G-Box represents drought-responsive element; W-Box represents WRKY binding site; LTR represents low-temperature-responsive element; WUN represents wound-responsive element; GARE and P-Box represent gibberellin-responsive elements; CGTCA motif and TGACG motif represent jasmonic acid-responsive elements; TCA-element represents salicylic-acid-responsive element, Figure S7: *MsNF-YB21* expression level in overexpression lines. Values are the means ± SE from three biological replicates. Significant differences were assessed using Student’s *t*-test in comparison to WT (Double asterisks denote *p* < 0.01), Figure S8: The phylogenetic tree of MsNF-YB21 and AtNF-Ys proteins was constructed using MEGA7 with the NJ method and a bootstrap of 1000 replicates. MsNF-YB21 was marked with red pot, Table S1: Basic information of 43 *MdNF-Y* members, Table S2: The approximate time prediction of duplication events among the identified *MdNF-Y* genes in apple, Table S3: Correlated values of *MdNF-Y* nucleotide sequences; Table S4. *Cis*-element analysis of *MdNF-Y* promoter sequences, Table S5: Expression level of *MsNF-Ys* in different tissues and under drought treatment, Table S6: RT-qPCR primers, Table S7: Primers for constructing vectors, Table S8: Accession Numbers.
