# Supplementary material for: Expression of the Malus sieversii NF-YB21 Encoded Gene Confers Tolerance to Osmotic Stresses in Arabidopsis thaliana
Source: Int J Mol Sci. 2021 Sep 10;22(18):9777. doi: 10.3390/ijms22189777 (PMC8467963; doi:10.3390/ijms22189777)
Supplement: Supplementary file 1 [file ijms-22-09777-s001.zip › Table S1 Basic information of 43 MdNF-Y members.pdf]

Table S1 Basic information of 43 *MdNF-Y* members

Name	Gene ID	Chromosome local	CDS length	No. of AA	pI	MW (Kda)	GRAVY	Subcellular localization prediction
NF-YA subunit								
MdNF-YA1	MD02G1086200	Chr2:6760401..6763923	927	308	8.552	33.93	-0.699	Nucleus.
MdNF-YA2	MD03G1174600	Chr3:23867258..23872067	1077	358	6.394	38.86	-0.994	Nucleus.
MdNF-YA3	MD05G1273300	Chr5:40831929..40836805	414	137	9.284	15.6	-1.088	Nucleus.
MdNF-YA4	MD07G1011300	Chr7:1023634..1029101	840	279	7.909	30.58	-0.905	Nucleus.
MdNF-YA5	MD09G1186200	Chr9:16163341..16168480	897	298	8.81	32.86	-0.619	Nucleus.
MdNF-YA6	MD10G1253300	Chr10:34584154..34588714	627	208	7.746	22.82	-0.963	Nucleus.
MdNF-YA7	MD11G1192400	Chr11:27332752..27339954	1047	348	7.711	37.92	-1.109	Nucleus.
MdNF-YA8	MD14G1041300	Chr14:3873166..3878129	930	309	9.05	33.94	-0.679	Nucleus.
MdNF-YA9	MD15G1213400	Chr15:17122823..17125801	903	300	8.63	33.21	-0.589	Nucleus.
NF-YB subunit								
MdNF-YB1	MD01G1112400	Chr1:22658861..22662399	645	214	8.11	22.93	-0.507	Nucleus.
MdNF-YB2	MD02G1191900	Chr2:17939911..17940645	735	244	5.136	27.02	-0.776	Nucleus.
MdNF-YB3	MD02G1192100	Chr2:18023631..18025409	1044	347	4.795	38.13	-0.482	Nucleus.
MdNF-YB4	MD03G1179600	Chr3:24691097..24691663	567	188	4.781	20.69	-0.428	Nucleus.
MdNF-YB5	MD03G1183400	Chr3:25025010..25025594	585	194	6.21	20.84	-0.837	Nucleus.
MdNF-YB6	MD03G1283700	Chr3:36413956..36414516	561	186	6.401	20.95	-0.895	Nucleus.
MdNF-YB7	MD04G1104700	Chr4:19216567..19220090	525	174	6.538	18.78	-0.884	Nucleus.
MdNF-YB8	MD04G1203300	Chr4:28953974..28954426	453	150	6.068	16.86	-0.749	Nucleus.
MdNF-YB9	MD05G1361000	Chr5:47685520..47685846	327	108	5.136	11.91	-0.694	Nucleus.
MdNF-YB10	MD05G1361600	Chr5:47696633..47698977	876	291	6.328	31.27	-0.627	Nucleus.
MdNF-YB11	MD07G1180200	Chr7:25950191..25953495	648	215	8.11	23.16	-0.541	Nucleus.
MdNF-YB12	MD09G1126900	Chr9:9795758..9796411	654	217	6.845	22.18	-0.735	Nucleus.
MdNF-YB13	MD10G1339000	Chr10:41496215..41496999	744	247	6.644	27.35	-0.601	Nucleus.

MdNF-YB14	MD11G1199300	Chr11:28769434..28769985	552	183	5.409	20.25	-0.667	Nucleus.
MdNF-YB15	MD11G1200400	Chr11:28876837..28877409	573	190	6.766	20.09	-0.684	Nucleus.
MdNF-YB16	MD11G1302500	Chr11:41788083..41788580	498	165	6.109	18.6	-0.92	Nucleus.
MdNF-YB17	MD12G1124800	Chr12:19970949..19980773	393	130	5.664	14.28	-0.874	Nucleus.
MdNF-YB18	MD12G1217100	Chr12:29460289..29460741	462	153	7.188	16.79	-0.727	Nucleus.
MdNF-YB19	MD13G1170200	Chr13:13815353..13815826	474	157	6.071	17.51	-0.91	Nucleus.
MdNF-YB20	MD15G1134600	Chr15:9778391..9779101	711	236	6.217	26.72	-1.01	Nucleus.
MdNF-YB21	MD15G1334200	Chr15:37152321..37152947	627	208	6.821	23.01	-0.86	Nucleus.
MdNF-YB22	MD17G1117200	Chr17:10167264..10168240	720	239	5.292	24.81	-0.65	Nucleus.
NF-YC subunit								
MdNF-YC1	MD02G1273400	Chr2:32804138..32809135	999	332	4.982	36.01	-0.704	Nucleus.
MdNF-YC2	MD04G1207500	Chr4:29269117..29271406	807	268	5.858	29.75	-0.578	Cytoplasm. Nucleus.
MdNF-YC3	MD05G1229600	Chr5:36298931..36300862	807	268	5.16	29.72	-0.478	Cytoplasm. Nucleus.
MdNF-YC4	MD06G1078500	Chr6:19362575..19362940	366	121	7.078	13.37	-0.161	Cytoplasm. Nucleus.
MdNF-YC5	MD06G1141200	Chr6:28495891..28498876	720	239	5.046	25.83	-0.409	Nucleus.
MdNF-YC6	MD07G1042300	Chr7:3543314..3555874	978	325	4.927	34.99	-0.683	Nucleus.
MdNF-YC7	MD10G1208800	Chr10:30806582..30809166	834	277	5.568	31.17	-0.636	Nucleus.
MdNF-YC8	MD12G1221800	Chr12:29861745..29864137	789	262	5.53	29.22	-0.617	Cytoplasm. Nucleus.
MdNF-YC9	MD13G1185200	Chr13:15766106..15768771	756	251	5.622	28.01	-0.643	Cytoplasm. Nucleus.
MdNF-YC10	MD14G1156400	Chr14:25090589..25093134	717	238	4.925	25.78	-0.405	Cytoplasm. Nucleus.
MdNF-YC11	MD15G1159400	Chr15:11916470..11917171	702	233	5.176	26.52	-0.157	Nucleus.
MdNF-YC12	MD16G1185900	Chr16:16034439..16037111	699	232	5.62	26.06	-0.673	Cytoplasm. Nucleus.

Notes:

As for the three subunit family members, the lengths of MdNF-YAs ranging from 137-358 AA (average length 282.78 AA) were the longest, the lengths of MdNF-YCs ranging from 121-332 AA (average length 253.83 AA) were shorter, the lengths of MdNF-YBs ranging from 108-347 AA (average length 201.64 AA) were the shortest. The predicted theoretical isoelectric points (pI) of the 43 MdNF-Ys ranged from 4.78 (MdNF-

YB4) to 9.28 (MdNF-YA3). The results of subcellular locations prediction showed that, all MdNF-YA and MdNF-YB proteins and part of MdNF-YC proteins (MdNF-YC1/5/6/7/11) were predicted to be nuclear proteins, while the rest of MdNF-YC proteins were predicted to be nuclear and cytosolic proteins (Table 1). The GRAVY score of MdNF-Y proteins were negative, suggesting that these proteins were hydrophilic.
