# Supplementary material for: Expression of the Malus sieversii NF-YB21 Encoded Gene Confers Tolerance to Osmotic Stresses in Arabidopsis thaliana
Source: Int J Mol Sci. 2021 Sep 10;22(18):9777. doi: 10.3390/ijms22189777 (PMC8467963; doi:10.3390/ijms22189777)
Supplement: Supplementary file 1 [file ijms-22-09777-s001.zip › Table S4 Cis-elements analysis of MdNF-Y promoter sequences.pdf]

Table S4 *Cis*-elements analysis of MdNF-Y promoter sequences

Gene name	ABRE	CGTCA-motif	DRE	GARE	LTR	MBS	P-Box	TCA-element	TGACG-motif	WUN	G-Box	W-Box
MdNF-YA1	1	0	2	0	0	1	0	1	0	0	1	0
MdNF-YA2	3	2	0	0	3	0	0	0	2	1	3	2
MdNF-YA3	0	1	0	0	1	0	0	1	1	0	0	2
MdNF-YA4	1	1	0	0	0	2	0	2	1	0	0	0
MdNF-YA5	4	0	0	1	0	1	1	3	0	2	4	0
MdNF-YA6	1	0	1	1	3	1	1	1	0	0	3	0
MdNF-YA7	5	2	0	0	2	2	0	0	2	1	5	1
MdNF-YA8	1	0	0	0	1	0	1	0	0	0	1	2
MdNF-YA9	3	0	0	1	0	1	0	0	0	0	4	0
MdNF-YB1	3	1	0	0	0	2	0	1	1	0	1	1
MdNF-YB2	2	0	0	1	0	0	0	0	0	3	2	0
MdNF-YB3	3	1	0	0	1	0	0	0	1	0	4	2
MdNF-YB4	3	0	0	0	0	0	1	2	0	1	4	1
MdNF-YB5	5	3	0	0	0	0	1	1	3	2	5	0
MdNF-YB6	5	0	0	0	0	0	0	0	0	0	5	0
MdNF-YB7	2	1	1	0	2	0	0	1	1	1	2	1
MdNF-YB8	8	4	1	0	0	0	0	0	4	1	7	1
MdNF-YB9	6	1	1	0	0	2	0	1	0	0	6	0
MdNF-YB10	5	1	0	0	1	0	1	0	1	0	6	1
MdNF-YB11	0	0	0	0	0	1	0	1	0	1	1	1
MdNF-YB12	6	1	0	0	0	2	0	1	1	0	9	1

MdNF-YB13	2	1	0	0	0	1	1	3	0	2	4	0
MdNF-YB14	2	0	0	0	0	0	1	0	0	0	3	0
MdNF-YB15	5	2	0	0	0	1	1	0	0	0	6	0
MdNF-YB16	7	2	0	0	0	0	1	0	2	0	8	0
MdNF-YB17	3	3	0	0	1	0	2	0	3	0	3	0
MdNF-YB18	6	0	0	0	0	1	0	1	0	2	4	1
MdNF-YB19	5	2	0	0	0	1	0	0	0	1	4	0
MdNF-YB20	2	2	3	0	0	0	1	3	0	0	5	2
MdNF-YB21	2	3	0	0	1	1	0	2	3	0	2	1
MdNF-YB22	1	0	0	0	1	1	0	0	2	1	3	1
MdNF-YC1	2	0	0	0	2	0	0	1	0	2	4	3
MdNF-YC2	1	1	0	0	2	0	0	0	1	1	1	0
MdNF-YC3	4	4	1	0	1	1	0	1	4	1	5	0
MdNF-YC4	8	3	0	0	1	1	0	0	3	2	11	1
MdNF-YC5	1	0	0	0	1	1	0	1	0	0	0	0
MdNF-YC6	7	2	0	0	2	1	0	0	2	1	8	2
MdNF-YC7	7	1	2	0	1	1	0	0	1	1	5	1
MdNF-YC8	1	1	0	0	2	0	0	0	1	0	1	0
MdNF-YC9	0	2	1	0	2	1	2	0	0	1	2	3
MdNF-YC10	5	1	0	1	1	0	1	2	0	0	10	1
MdNF-YC11	3	0	1	0	0	1	0	0	0	3	4	2
MdNF-YC12	0	1	0	1	1	2	2	0	1	0	0	1

Notes: MBS represents MYB binding site; ABRE represents abscisic acid (ABA) responsive element; DRE represents dehydration responsive element; G-Box represent drought responsive element; W-Box represents WRKY binding site; LTR represents low temperature responsive element; WUN represents wound responsive element; GARE and P-Box represent gibberellin responsive elements; CGTCA motif and TGACG motif represent jasmonate acid responsive elements; TCA-element represents salicylic acid responsive element. In these *cis*-elements, five were reported related with the drought stress, including ABREs, MBSs, G-Boxs, W-Boxs and DREs (colored with red).
