# Supplementary material for: Expression of the Malus sieversii NF-YB21 Encoded Gene Confers Tolerance to Osmotic Stresses in Arabidopsis thaliana
Source: Int J Mol Sci. 2021 Sep 10;22(18):9777. doi: 10.3390/ijms22189777 (PMC8467963; doi:10.3390/ijms22189777)
Supplement: Supplementary file 1 [file ijms-22-09777-s001.zip › Table S6 RT-qPCR primers.pdf]

Table S6 RT-qPCR primers

Gene	Forward primer(5' to 3')	Reverse primer(5' to 3')
MdNF-YA1	TGCTTCTAACAAAATGGTGGCAT	GGCGCAGTGCTTAGCTGATT
MdNF-YA2	CCTACAGTGCGTGATGATTGC	AGAAAAGGAGGATAACCCAAAGGT
MdNF-YA3	TTCTGTGCGCCATCATTCTG	CGGGATGAGACATTCCAATTGC
MdNF-YA4	ACACTCTAATGGTAATGGCAACGAT	TCGTAGAGGGACCTCATTTACTGG
MdNF-YA5	CAAATTGATGTTAGCCGTTCAATTGG	GGAGTTATCCCCACCACCTG
MdNF-YA6	TTTGCATGAATCTCGACACCAG	TCATCCGGTTGGTTCCCA
MdNF-YA7	AGCTTATGGACATCAGCCTTTG	GTCTTCGCCTTAGAATCCCTTG
MdNF-YA8	ACCAGGGAACAGCCCAATA	GCTGAATCCAAGCTCAAAATG
MdNF-YA9	CCACAAGGTTATCATGACGCA	TCCCATCAGCACCCTTACCA
MdNF-YB1	CGGTGTTGATAAATGGTAGTAAGCA	GAGAGACAGAGGGGAAGGTGAA
MdNF-YB2	GTCGGGTGGTCCTCAACGA	GTTGGCTATCGGCATGTATCG
MdNF-YB3	TCCGAGTACATCGCCTTCATAACC	AGTTGTCAAACCCGAGCTTTCC
MdNF-YB4	TAACTGCTGAAGCCAACCAG	AGCGATGGAGGAAAACGTTG
MdNF-YB5	TACGTGGAGCCTCTGAAGGTT	CATACCCAACCCCACCATTCCC
MdNF-YB6	CTGAAGTTTCACCACCAAGGAC	GAGAGCGAGCTATTTCCCCTT
MdNF-YB7	CCAGGAATGCGTTTCTGAGTTC	AAAGTTGCCATTGCCCAGAG
MdNF-YB8	ACAACAACCAAAACACGGCAA	TCTCAAGCACCCGAAACTCC
MdNF-YB9	TTGCAAACGTGAGCAGGATC	TGAAGCTGATGAACTCGGAGAC
MdNF-YB10	AAGGAAACCATCCAAGAGTGCG	TGCAGGTAGAGGGTGAGTGGC
MdNF-YB11	TCACCAGCGAGGCAAGCG	ACCCTCCCTGTACCTACTCAAGTAAATC
MdNF-YB12	CAACGGCGACGATTTGCTAT	GCCCTCCATCTCCCGATACTT
MdNF-YB13	GGAAACCATCCAAGAGTGCGT	TGCAGGTAGAGGGTGAGTGGC
MdNF-YB14	GTCCGAGTACATCGCCTTCATAAC	GTAATTGTCAAACCCGAGCTTTCC
MdNF-YB15	ACGGCGACGATTTGCTCTG	CTTCTCCCCTTCCATCTCCCTA
MdNF-YB16	CGATGATTACGCTGAGCCAC	GTCCTTGGTGGCAGTTTGTTC
MdNF-YB17	CTAAGAAAGATGTTCAGCCAAGTCC	CATCTCAAACGGGCAGCTAAATT
MdNF-YB18	TGCTCCCAATTGCAAACGTG	AGCCTCGCCAGTTACAAAAC
MdNF-YB19	AAAGGATGATGATATTGATGGAGGC	TCTTGGCGTTGGGTGGC
MdNF-YB20	AACTGTCCAAGAATGCGTCTCC	GTTGTGATAGCCCAAATGATGTCC
MdNF-YB21	GCCAGAGAAGAAGACCACCC	CTGTCGTCCCCCAACTGAAT
MdNF-YB22	CAGGAGTGCGTGTCGGAG	GTGGTCATCGCCCATAGCAAATC
MdNF-YC1	CCATGAGACTGAATCTGAACCCT	CATCATCCGCCACCATTCCA
MdNF-YC2	GCCCACCGCCTGTTTG	CAGACTGCATAGCTCCCACTG
MdNF-YC3	CAAGTTCTGGCCAAATGGCATCA	CTGCGATGGGGAAGTGACG
MdNF-YC4	CCACCCTCGAAGGGAAGAGG	AGCACGACCCCGAAACCAA
MdNF-YC5	CTACCCAGCAACCCAATCCT	CGGCAGCTGGTGATTCTTGA
MdNF-YC6	ATACCCGTTTTCCTGCTGCC	TGCAAGAATAGCTCCAACGC
MdNF-YC7	CAGTTGGTGTCGTAGCAGGTT	CTGATGTGACGGATCAGGGA
MdNF-YC8	AAACCCAAGAACCTCCCAGAA	CGACTCGGAAACATCCATCT
MdNF-YC9	GTGGGAAAGCCTGTGATGGA	TTGTTGCTGTGGCCATGTTG
MdNF-YC10	TACCCAGCAACCCAATCCTC	CTCTCAATCTCCTGGCGCTG
MdNF-YC11	ATTTCTTCGCCCTGCACTGT	GTCATGTGGGAAGTGTGGGA

MdNF-YC12	TGCATCAATCCCAAGAGGTGG	TATTCTGATTCCCCGGAGCAC
Histone H3	TGAAGAAGCCCCACAGATA	GTCAGTCTTGAAGTCCTGCG
AtFSD1	CCTCAAGAAACAGGTTCTTGGAACCG	GGTCATGAATGTCTTTATGTAATC
AtFSD2	GGAACCGCATATGAGCCGGGAAACC	CTTGTCTTCCTCCTTTGGGAGAGG
AtFSD3	GGTGGTTTAAAGGTTGAAGCTTACTACGG	CCTTATCAATCTGCTCAAGAACACCC
AtCSD1	TGGTAGTTGTGTGTATCTTCTGGTGTGTG	TGAAAGTACCATATCCAGGACTCTTGTTACC
AtCSD2	GCAGCAGCCATGGCTGCCACC	GGCCCTGGAGTGAGACCAGTG
AtCSD3	CGGGAGGGCGGTTGTTGTGCATGCGG	CTGAGTGTGGCTCTGTCCGTTGATCTCAAAAGCTAT
AtMSD	CCACCAAAAGGATCTCTTGGTAGTGCC	CCTCGCTTGCATATTTCCAGTTGATCAC
AtCAT1	AGGAGCCAATCACAGCC	TCAAGACCAAGCGACCA
AtCAT2	AACTCCGCCTGCTGTCTG	ATAGGGCATCAATCCATC
AtCAT3	TCACAGCCACGCCACTAA	AGAACCAAGCGACCAACC
AtPER12	CCTCAACATCACCGACTTAGTA	CTCCTTATGTCATTCACTTGCG
AtPER34	ATCGTCCTTCTGATCTCGTTGCT	GATCAAAATCTACCAAGGCACTTC
AtPER42	GAAGAACTCACTGTGTCAAGCT	AACCCTTGTTGTCTAGGATGTT
AtPER51	ATTCTCTCTCTTTTTCTCGCCATCA	AAGTGCATTGAGTTGGTTGAGATCA
AtACT2	CTCCTTTGTTGCTGTTGACTAC	GCACAATGTTACCGTACAGATC
