# Supplementary material for: Expression of the Malus sieversii NF-YB21 Encoded Gene Confers Tolerance to Osmotic Stresses in Arabidopsis thaliana
Source: Int J Mol Sci. 2021 Sep 10;22(18):9777. doi: 10.3390/ijms22189777 (PMC8467963; doi:10.3390/ijms22189777)
Supplement: Supplementary file 1 [file ijms-22-09777-s001.zip › Table S7 Primers for constructing vectors.pdf]

Table S7 Primers for constructing vectors

Gene	Forward primer(5' to 3')	Reverse primer(5' to 3')
MsNF-YB21	ATGAGTGGAAAAAGAAATCAAACGTGT	CTACCATCCTATCCCGTTATGAATATGATG
MdNF-YC8	ATGGATCCGCAAGGGCATA	CTAATGATCTGATGGCGACTGC
BD-MsNF-YB21	TGATCTCAGAGGAGGACCTGCATATGATGAGTGG AAAAAGAAATCAAACGTGT	TAGTTATGCGGCCGCTGCAGGTCGACGCCAT CCTATCCCGTTATGAATATGATG
AD-MsNF-YC8	ACGACGTACCAGATTACGCTCATATGATGGATCCG CAAGGGCATA	ATCTGCAGCTCGAGCTCGATGGATCCCATGA TCTGATGGCGACTGC
BiFC-MsNF-YB21	GGATCCATCGATAGTACTGTCGACATGAGTGGAA AAAGAAATCAAACGTGT	TTGCTCCATCCCGGGAGCGGTACCCCATCCT ATCCCGTTATGAATATGATG
BiFC-MsNF-YC8	GGATCCATCGATAGTACTGTCGACATGGATCCGCA AGGGCATA	TGGGTACATCCCGGGAGCGGTACCATGATCT GATGGCGACTGC
GFP-MsNF-YB21	ACGGGGGACTCTTGACCCATGGTAATGAGTGGAA AAAGAAATCAAACGTGT	TTCTCCTTTACTAGTCAGATCTACCCATCCTA TCCCGTTATGAATATGATG
