# Supplementary figures and images for: Expression of the Malus sieversii NF-YB21 Encoded Gene Confers Tolerance to Osmotic Stresses in Arabidopsis thaliana
Source: Int J Mol Sci. 2021 Sep 10;22(18):9777. doi: 10.3390/ijms22189777 (PMC8467963; doi:10.3390/ijms22189777)

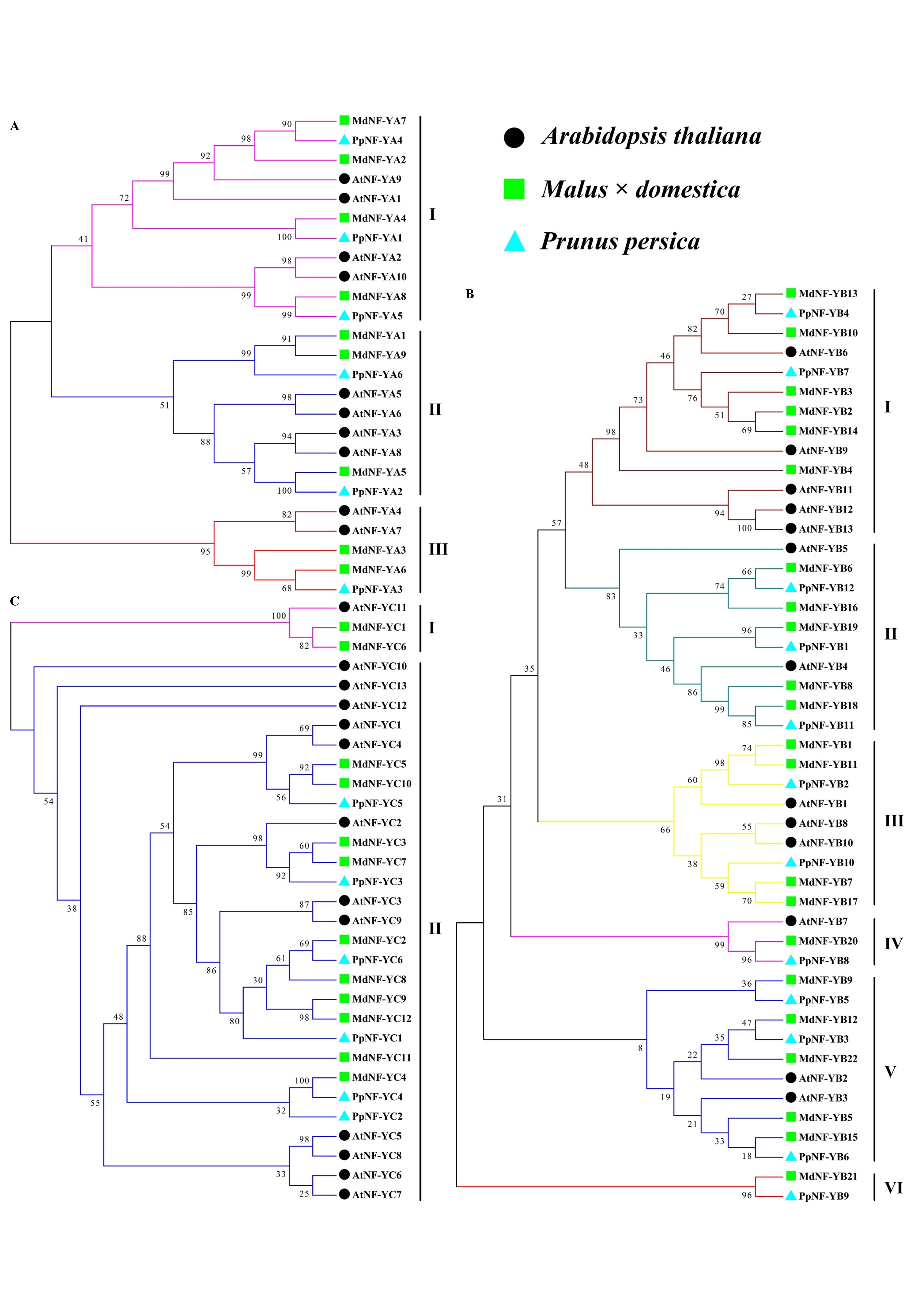

Supplement: Supplementary file 1 [file ijms-22-09777-s001.zip › Figure S1.jpg]

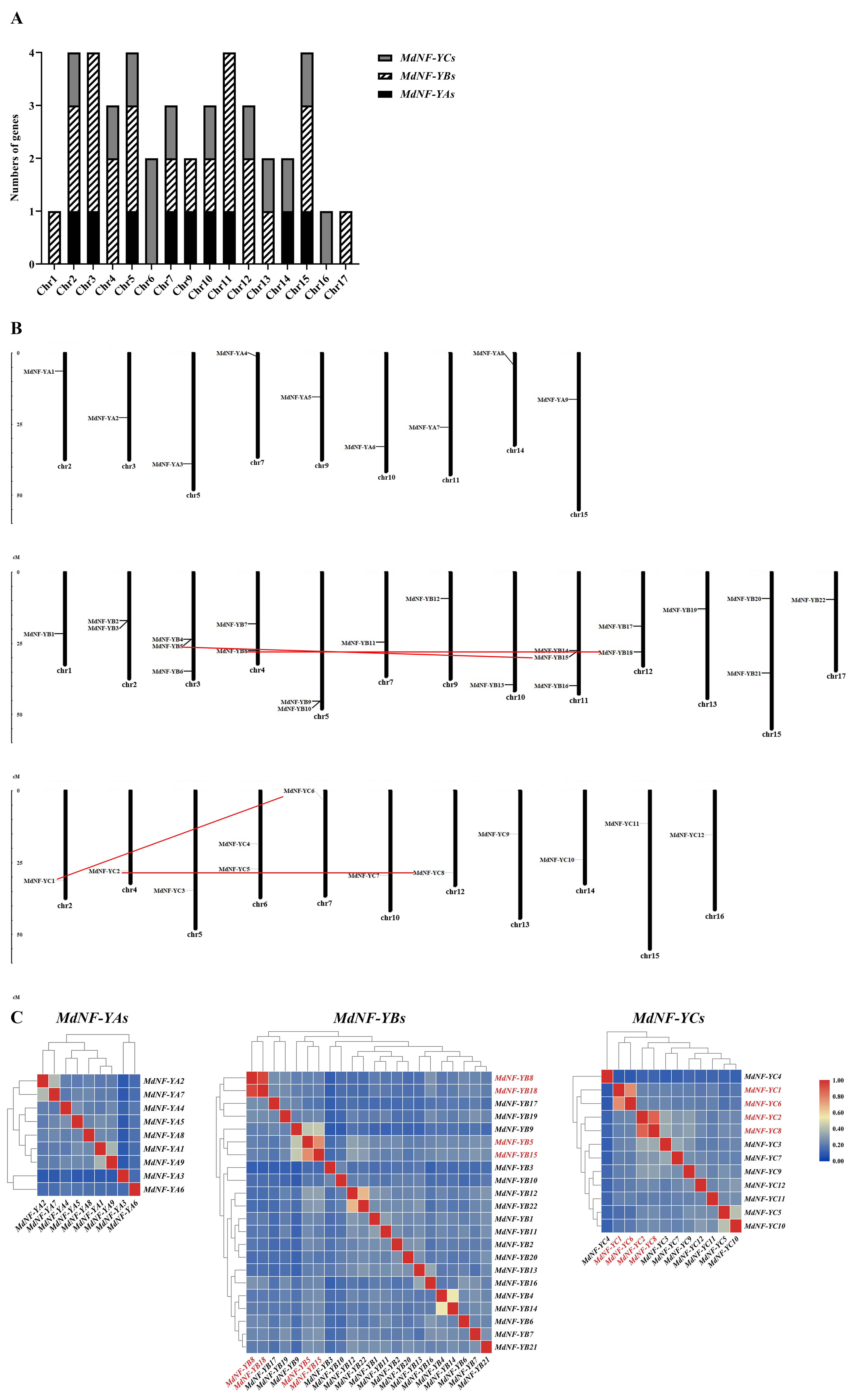

Supplement: Supplementary file 1 [file ijms-22-09777-s001.zip › Figure S2.jpg]

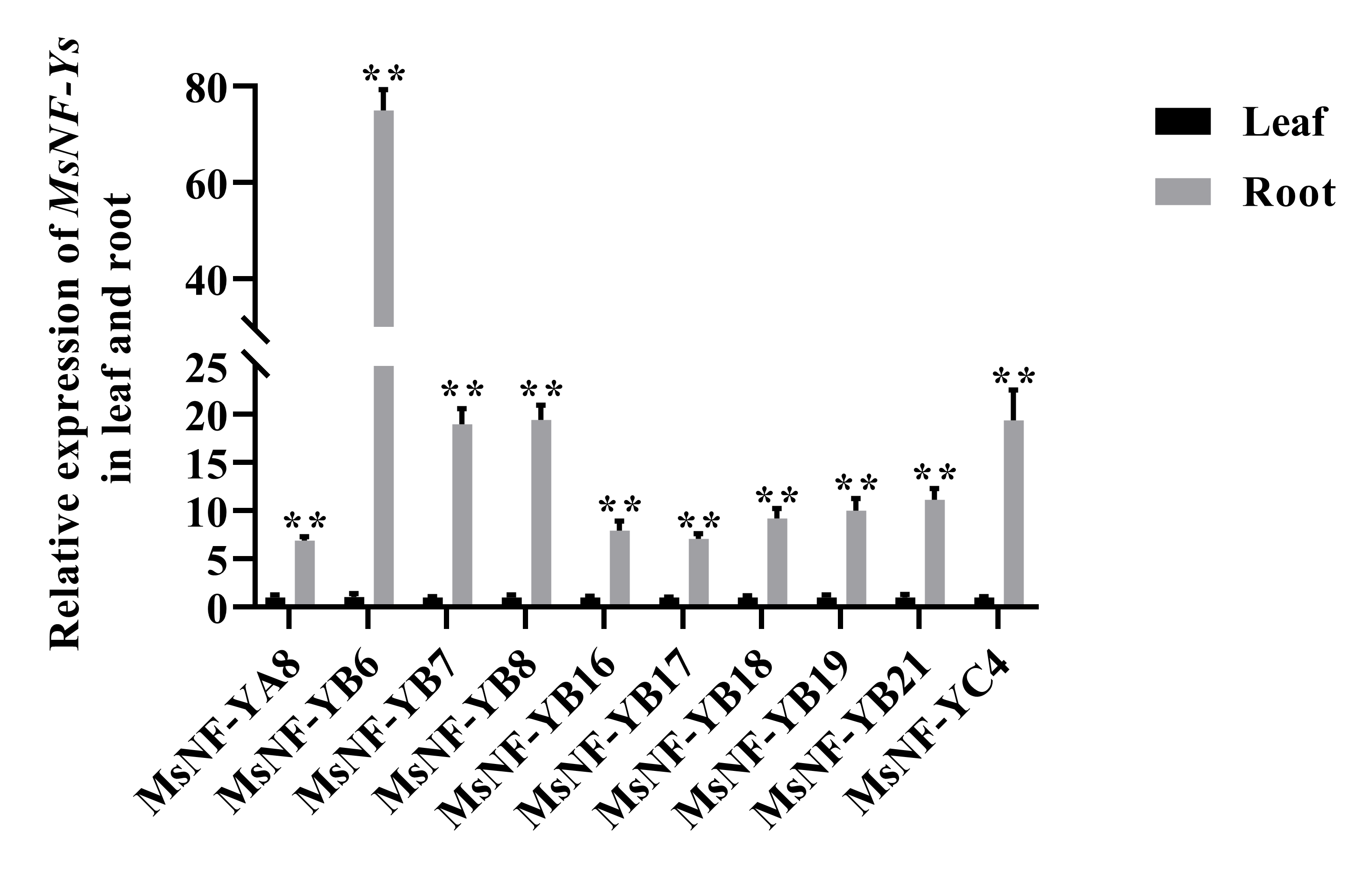

Supplement: Supplementary file 1 [file ijms-22-09777-s001.zip › Figure S3.tif]

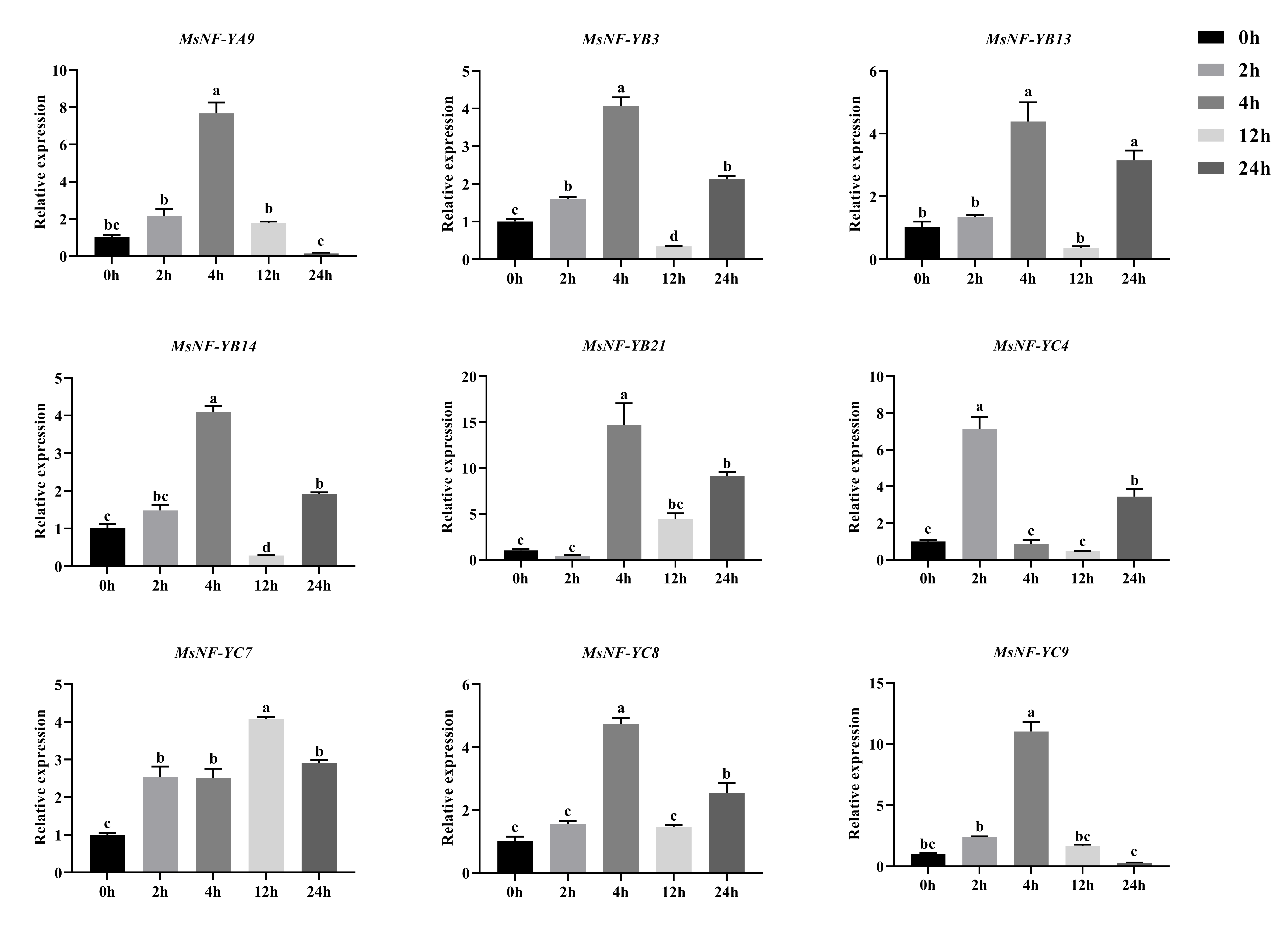

Supplement: Supplementary file 1 [file ijms-22-09777-s001.zip › Figure S4.tif]

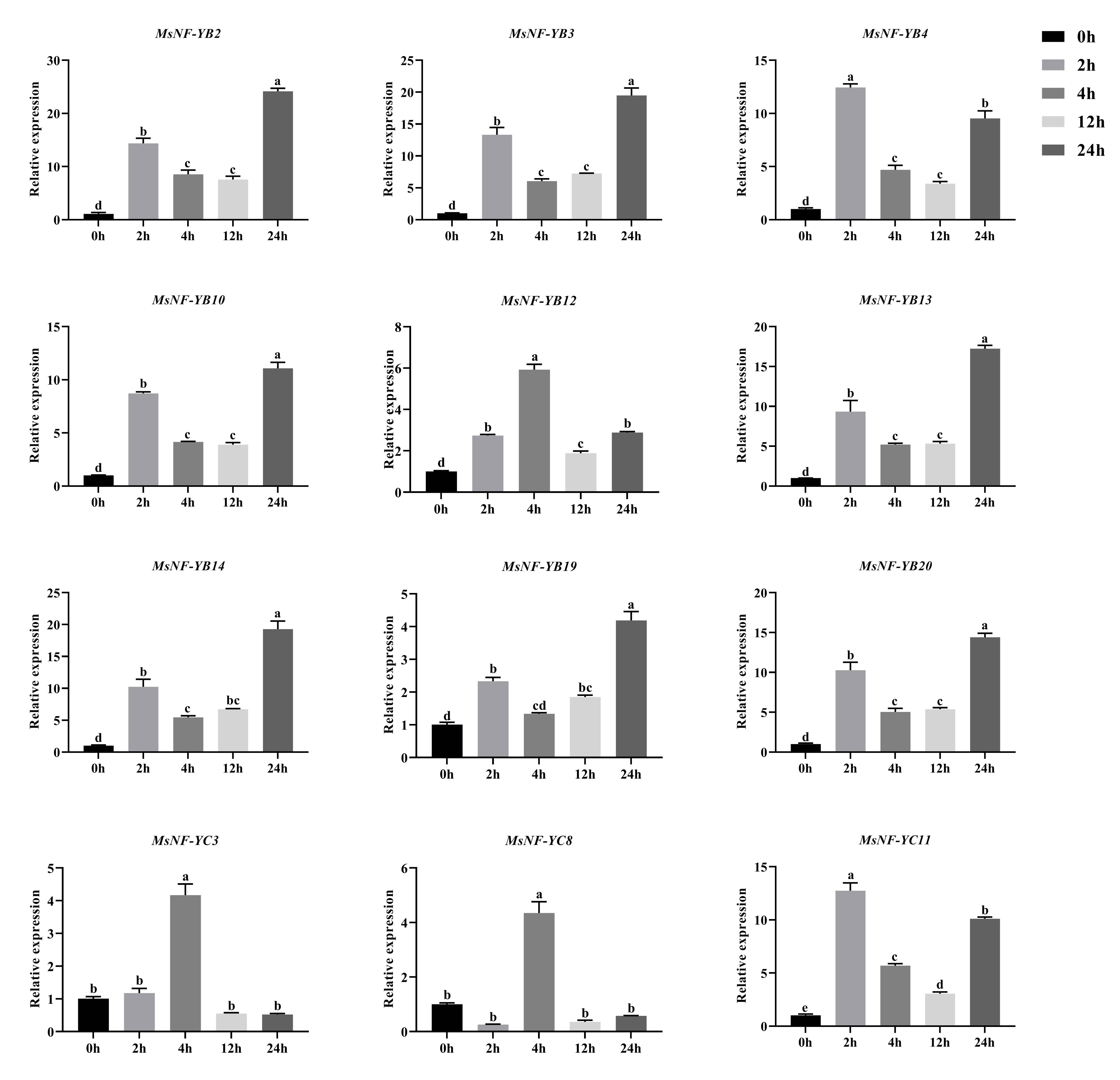

Supplement: Supplementary file 1 [file ijms-22-09777-s001.zip › Figure S5.jpg]

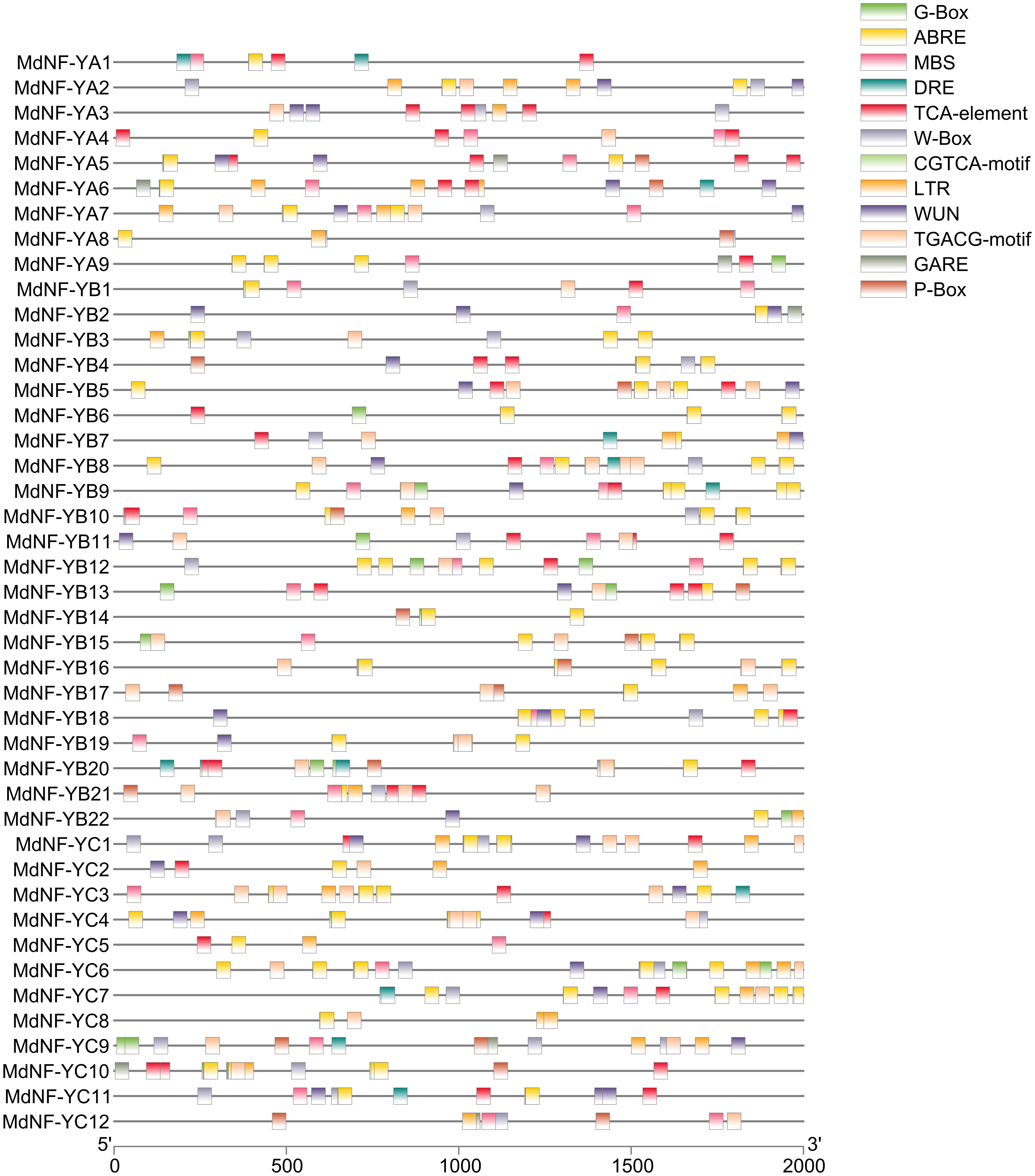

Supplement: Supplementary file 1 [file ijms-22-09777-s001.zip › Figure S6.jpg]

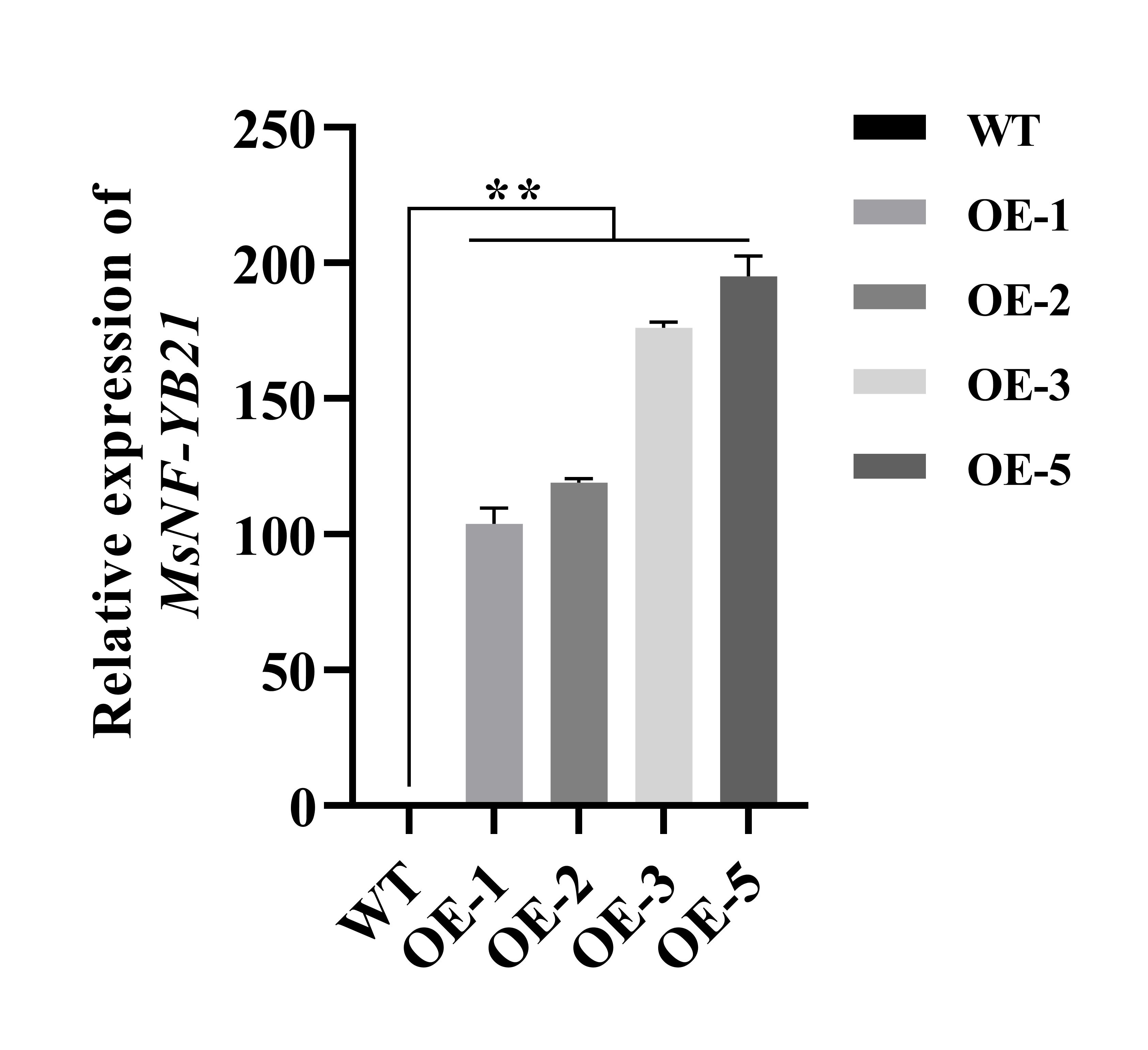

Supplement: Supplementary file 1 [file ijms-22-09777-s001.zip › Figure S7.tif]

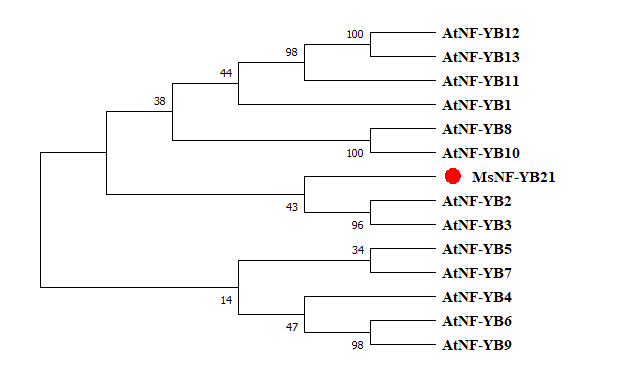

Supplement: Supplementary file 1 [file ijms-22-09777-s001.zip › Figure S8.tif]
